# Statistical Ring Opening Metathesis Copolymerization of Norbornene and Cyclopentene by Grubbs’ 1st-Generation Catalyst

**DOI:** 10.3390/molecules200915597

**Published:** 2015-08-27

**Authors:** Christiana Nikovia, Andreas-Philippos Maroudas, Panagiotis Goulis, Dionysios Tzimis, Patrina Paraskevopoulou, Marinos Pitsikalis

**Affiliations:** 1 Industrial Chemistry Laboratory, Department of Chemistry, University of Athens, Panepistimiopolis Zografou, Athens 15771, Greece; E-Mails: xristiana2309@gmail.com (C.N.); andreasmaroudas@hotmail.com (A.-P.M.); one_nosense@hotmail.com (P.G.); tzimisd@yahoo.com (D.T.); 2 Inorganic Chemistry Laboratory, Department of Chemistry, University of Athens, Panepistimiopolis Zografou, Athens 15771, Greece; E-Mail: paraskevopoulou@chem.uoa.gr

**Keywords:** Ring Opening Metathesis Polymerization (ROMP), norbornene, cyclopentene, copolymerization, thermogravimetric analysis (TGA)

## Abstract

Statistical copolymers of norbornene (NBE) with cyclopentene (CP) were prepared by ring-opening metathesis polymerization, employing the 1st-generation Grubbs’ catalyst, in the presence or absence of triphenylphosphine, PPh_3_. The reactivity ratios were estimated using the Finemann-Ross, inverted Finemann-Ross, and Kelen-Tüdos graphical methods, along with the computer program COPOINT, which evaluates the parameters of binary copolymerizations from comonomer/copolymer composition data by integrating a given copolymerization equation in its differential form. Structural parameters of the copolymers were obtained by calculating the dyad sequence fractions and the mean sequence length, which were derived using the monomer reactivity ratios. The kinetics of thermal decomposition of the copolymers along with the respective homopolymers was studied by thermogravimetric analysis within the framework of the Ozawa-Flynn-Wall and Kissinger methodologies. Finally, the effect of triphenylphosphine on the kinetics of copolymerization, the reactivity ratios, and the kinetics of thermal decomposition were examined.

## 1. Introduction

Ring Opening Metathesis Polymerization (ROMP), a relatively new tool in the field of polymer chemistry, has emerged as a powerful and broadly-applicable method for synthesizing macromolecular materials. ROMP is a chain growth polymerization process where cyclic olefins are converted to a polymeric material [1,2]. The mechanism of the polymerization is based on olefin metathesis, a unique metal-mediated carbon-carbon double bond exchange process [3,4,5,6,7,8,9]. Initiation begins with coordination of a cyclic olefin to a transition metal alkylidene complex. Subsequent [2 + 2]-cycloaddition affords a four-membered metallacyclobutane intermediate which effectively initiates the polymerization reaction. This intermediate undergoes a cycloreversion reaction to afford a new metal alkylidene. Although the resulting complex has increased in size (due to the incorporated monomer), its reactivity toward cyclic olefins is similar to the initiator. Hence, analogous steps are repeated during the propagation step until polymerization ceases (*i.e*., all monomer is consumed, a reaction equilibrium is reached, or the reaction is terminated). Living ROMP reactions are commonly quenched deliberately through the addition of a specialized reagent (Scheme S1 in the Supporting Information Section (SI)). The function of this reagent is to (1) selectively remove and deactivate the transition metal from the end of the growing polymer chain and (2) install a known functional group in place of the metal [10].

Apart from homopolymer synthesis, ROMP reactions have also been utilized in the challenging field of copolymerization. It is known that copolymerization is the most successful and powerful method for effecting systematic changes in polymer properties [11]. The incorporation of two different monomers, with diverse physical and/or chemical properties, in the same polymer molecule in varying proportions leads to the formation of new materials with great scientific and commercial importance [12]. The most important feature of successful copolymerization is the ability to control the amount and distribution of the comonomers in the product [13,14,15,16,17,18,19]. The elucidation of copolymer structure (copolymer composition, monomer sequence distribution) and kinetics (propagation rate coefficients) are the major concerns for the prediction of copolymer properties and the correlation between structure and properties [20]. The chemical composition of the copolymers depends on the degree of incorporation of the comonomers and the relative reactivity between them. Monomer reactivity ratios are very important parameters for the elucidation of copolymer structure and kinetics [21,22].

A wide range of catalytic systems have been developed for the ROMP reaction, based mainly on mononuclear transition metal complexes across the Periodic Table (Ti, Nb, Ta, Cr, Mo, W, Re, Co, Ru, Os), including uni-, bi-, ternary, quaternary, and multicomponent systems [23,24,25,26,27,28]. In recent years, catalyst development for “living” ROMP has focused on ruthenium-based initiators, which are more selective for olefins, and are therefore more tolerant to functionalities in the reaction system, thus expanding the range of monomers which can be employed [29]. Grubbs’ catalysts are more accessible, compatible with a wide range of solvents, and tolerant to ubiquitous impurities such as oxygen and water [30]. For these reasons, they have found extensive use in organic and polymer chemistry and afford polymers with novel mechanical, electronic and, more recently, biological properties [31].

Apart from studies involving the use of these catalysts in various ROMP reactions, tremendous effort has been simultaneously directed toward understanding the mechanism of these Ru catalysts. It was found that in Ru-based ROMP the mechanism of the olefin metathesis reaction was dissociative in nature. Thus, a phosphine ligand must dissociate from the catalyst before the olefin coordination to generate the 14-electron intermediate. This intermediate can be trapped by free phosphine to regenerate the initial alkylidene or can bind substrate and undergo metathesis (Supplementary Scheme S2 in SI) [32]. In essence, by adding excess phosphine to the solution, the binding equilibrium between Ru and phosphine ligand is shifted towards the inactive bound state, thereby slowing the propagation and the initiation reactions. However, the decrease in propagation rate is much more pronounced than initiation. It was found that the ratio of the initiation over the propagation rate constants, k_i_/k_p_, is equal to 10.2 when the polymerization takes place in the presence of excess PPh_3_ (5 fold molar excess over the catalyst), whereas k_i_/k_p_ equals 0.73 in the absence of excess PPh_3_. Under these conditions a better controlled polymerization is promoted. Indeed, recent studies have shown that addition of phosphine during ROMP polymerization results in polymers with narrow molecular weight distribution [33].

In this study, we report the synthesis of statistical copolymers of norbornene, NBE, and cyclopentene, CP, via ROMP catalyzed by Grubbs’ catalyst. This copolymerization reaction has been studied in the past employing various catalytic species [34,35,36,37,38,39,40]. It was soon realized that NBE is much more reactive than CP, due to the difference in strain energies of the two monomers. Several researchers tried to use different catalysts promoting the higher incorporation of CP units in the copolymeric structures. In older studies employing non-well characterized catalysts the reactivity ratios were determined confirming the difference in reactivity of these monomers. However, in more recent works the monomer reactivity ratios, the mean sequence lengths and the monomer dyad distributions have not been determined. In the present study we report a detailed analysis of the copolymerization behavior of NBE and CP and in addition we examine the effect of the presence of an excess of PPh_3_ in the copolymerization mixture on the reactivity ratios and the kinetics of copolymerization. The kinetics of thermal decomposition of the copolymers was also investigated and compared with the respective homopolymers.

## 2. Results and Discussion

### 2.1. Statistical Copolymers of NBE with CP in the Absence of Triphenylphosphine

The ROMP copolymerization of NBE with CP was conducted in CH_2_Cl_2_ solutions at 0 °C (Scheme S3). The copolymerizations were allowed to proceed to low conversion (less than 20%, except for sample 80/20 for which the conversion was 39%), in all cases, satisfying the differential copolymerization equation. The molecular characteristics of the samples are given in Table 1. Different copolymers are symbolized by the various feed molar ratios of the monomers; for example, sample 20/80 indicates the copolymer for the synthesis where 20% NBE and 80% CP were employed as the molar feed composition. The molecular weights were measured by SEC using a calibration curve constructed by polystyrene standards. It is obvious that rather high molecular weight copolymers of relatively broad molecular weight distributions were obtained in agreement with previous results describing the homopolymerization of NBE and CP under similar experimental conditions [41]. The SEC traces of the copolymers and a characteristic ^1^H-NMR spectrum are given in the SI (Figures S1 and S2).

**Table 1:** Molecular characteristics of the P(NBE-*co*-CP) copolymers synthesized in the absence ^a^ or presence ^b^ of PPh_3_.

Sample	M_w_ × 10^−3^ Daltons	M_w_/M_n_^c^	% mol NBE ^d^	% mol CP ^d^
20/80	637.5	1.6	49	51
40/60	615.8	1.8	57	43
50/50	101.1	1.7	63	37
60/40	161.5	1.9	67	33
80/20	127.1	1.8	72	28
20/80P	58.4	1.5	50	50
40/60P	65.7	1.7	62	38
50/50P	78.2	1.5	64	36
60/40P	101.4	1.2	69	31
80/20P	134.3	1.2	74	26

^a^ Conditions: solvent CH_2_Cl_2_/0 °C; ^b^ Conditions: solvent CH_2_Cl_2_/25 °C; ^c^ by SEC in THF; ^d^ by ^1^H-NMR.

^1^H-NMR spectroscopy was employed for the structural characterization of the samples and for the calculation of the copolymer composition. The composition was calculated by integrating the areas at 2.2–2.9 ppm (H^6^ and H^10^ PNBE) and 1.7–2.1 ppm (H^8^ PNBE and H^3,5^ PCP).

#### 2.1.1. Monomer Reactivity Ratios and Statistical Analysis of the Copolymers

The monomer reactivity ratios were determined using the Finemann-Ross (FR) [42], inverted Finemann-Ross (IFR) [42], and Kelen-Tüdos (KT) [43] graphical methods. A computer program, called COPOINT, was also employed [44]. According to the Finemann-Ross method, the monomer reactivity ratios can be obtained by the equation:

G = H r_NBE_ − r_CP_ (1)

where the reactivity ratios, r_NBE_ and r_CP_ correspond to the NBE and CP monomers, respectively. The parameters G and H are defined as follows:

G = X(Y − 1)/Y and H = X^2^/Y (2)

with

X = M_NBE_/M_CP_ and Y = dM_NBE_/dM_CP_ (3)

M_NBE_ and M_CP_ are the monomer molar compositions in feed and dM_NBE_ and dM_CP_ the copolymer molar compositions.

The inverted Finemann-Ross method is based on the equation:

G/H = r_NBE_ − (1/H)r_CP_ (4)

The plots of the G *vs.* H values and the G/H *vs.* 1/H values yield the reactivity ratios r_NBE_ and r_CP_ from the intercept and the slope of the graphs.

Alternatively, the reactivity ratios can be obtained using the Kelen-Tüdos method which is based on the equation:

η = (r_NBE_ + r_CP_/α)ξ − r_CP_/α (5)

where η and ξ are functions of the parameters G and H:

η = G/(α + H) and ξ = H/(α + H)
(6)

and α a constant which is equal to (H_max_·H_min_)^1/2^, H_max_, H_min_ being the maximum and the minimum H values, respectively, from the series of measurements. From the linear plot of η as a function of ξ, the values of η for ξ = 0 and ξ = 1 are used to calculate the reactivity ratios according to the equations:

ξ = 0 ⇒ η = −r_CP_/α and ξ = 1 ⇒ η = r_NBE_ (7)

**Figure 1:**
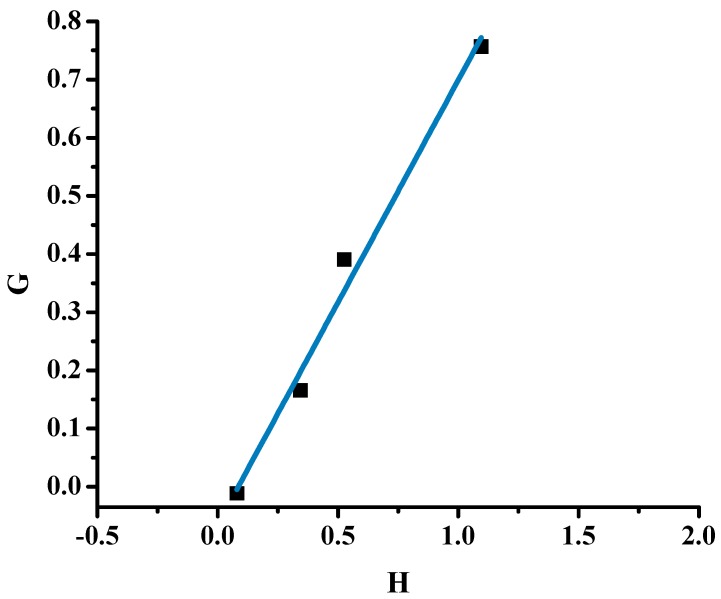
FR plot of the statistical copolymers.

The copolymerization data for all samples are provided in Table 2. The sample 80/20 was not taken into account for the calculation of the reactivity ratios, since the conversion of the polymerization reaction was much higher than the other samples, thus deviating from the copolymerization equation. The FR graphical plot is given in Figure 1, whereas the other plots concerning the methods previously reported are given in Figures S3 and S4 in the SI, whereas the reactivity ratios are summarized in Table 3. All plots for the different graphical methods were linear, thus indicating that these reactions follow conventional copolymerization kinetics and that the reactivity of the active polymerization chain end is determined only by the terminal monomer unit.

The computer program COPOINT evaluates the copolymerization parameters using comonomer/copolymer composition data as obtained from copolymerization experiments with finite monomer conversion. Although the mathematical treatment can be applied up to full monomer conversion, it is recommended not to exceed 20–30 mol %. COPOINT numerically integrates a given copolymerization equation in its differential from. The copolymerization parameters can be obtained by minimizing the sum of square differences between measured and calculated polymer compositions. Errors of the fitted parameters are estimated from the statistical error of the sum of square differences, as well as from a quadratic approximation of this sum in the vicinity of the optimized values of the copolymerization parameters.

**Table 2:** Copolymerization data for the copolymers.

Sample	X	Y	H	G	η	ξ
20/80	0.277	0.960	0.080	−0.011	−0.030	0.212
40/60	0.675	1.325	0.344	0.166	0.259	0.538
50/50	0.946	1.703	0.525	0.390	0.475	0.640
60/40	1.491	2.030	1.095	0.757	0.544	0.788
α = 0.295						
20/80P	0.250	1.000	0.062	0	0	0.199
40/60P	0.667	1.631	0.272	0.258	0.493	0.520
50/50P	1.000	1.778	0.562	0.437	0.537	0.691
60/40P	1.500	2.226	1.011	0.826	0.654	0.801
α = 0.251						

**Table 3:** Reactivity ratios.

Method	r_NBE_	r_CP_	r_NBE_	r_CP_
	in the Absence of PPh_3_		in the Presence of PPh_3_	
F-R	0.76 ± 0.06	0.06 ± 0.003	0.84 ± 0.06	0.02 ± 0.002
i F-R	0.78 ± 0.07	0.07 ± 0.004	0.96 ± 0.12	0.06 ± 0.004
KT	0.82 ± 0.10	0.07 ± 0.005	0.87 ± 0.07	0.02 ± 0.004
COPOINT	0.77 ± 0.14	0.02 ± 0.001	0.96 ± 0.23	0.03 ± 0.001
**Other catalytic systems ^a^**				
IrCl_3_/1,5-COD	5.6	0.07		
WCl_6_/EtAlCl_2_	13	0.32		
WCl_6_/Ph_4_Sn	2.6	0.55		
WCl_6_/Bu_4_Sn	12	0.27		
WCl_6_/Ph_4_Sn/EAc	2.2	0.62		

^a^: From ref. [34].

It is obvious that all methods provide similar data concerning the reactivity ratios for both monomers. According to the data obtained by the Kelen-Tüdos method r_NBE_ = 0.82 and r_CP_ = 0.07. These results imply that the rate of NBE incorporation into the copolymer structure is much higher than the rate of CP incorporation and is in agreement with the much higher rate of NBE homopolymerization compared to the rate of CP homopolymerization in ROMP procedures [45,46,47,48,49,50]. This case is referred to as a non-ideal non-azeotropic copolymerization [51]. Previous studies employing catalysts based on Ir and W provided similar results in terms of the reactivity ratios [34,35,36]. However, the difference in reactivity was even higher than in the present study. Grubbs’ catalysts in the presence of MoCl_5_ or WCl_6_, RuCl_3_-phenol and modified Grubbs’ catalysts have been previously employed to provide alternating PNBE/PCP copolymers [37,38,39,40]. This is direct evidence that the catalytic system greatly influences the reactivity ratios of the two monomers.

The statistical distribution of the dyad monomer sequences M_NBE_-M_NBE_, M_CP_-M_CP_, and M_NBE_-M_CP_ were calculated using the method proposed by Igarashi [52]:

(8) $$X={\text{φ}}_{NBE}-\frac{2{\text{φ}}_{NBE}\left(1-{\text{φ}}_{NBE\;}\right)}{1+{\left[{\left(2{\text{φ}}_{NBE\;}-1\right)}^{2}+4r_{NBE}r_{CP}{\text{φ}}_{NBE}\left(1-{\text{φ}}_{NBE}\right)\right]}^{1/2}}$$

(9) $$Y=\left(1-{\text{φ}}_{NBE}\right)-\frac{2{\text{φ}}_{NBE}\left(1-{\text{φ}}_{NBE\;}\right)}{1+{\left[{\left(2{\text{φ}}_{NBE\;}-1\right)}^{2}+4r_{NBE}r_{CP}{\text{φ}}_{NBE}\left(1-{\text{φ}}_{NBE}\right)\right]}^{1/2}}$$

(10) $$Z=\frac{4{\text{φ}}_{NBE}\left(1-{\text{φ}}_{NBE\;}\right)}{1+{\left[{\left(2{\text{φ}}_{NBE\;}-1\right)}^{2}+4r_{NBE}r_{CP}{\text{φ}}_{NBE}\left(1-{\text{φ}}_{NBE}\right)\right]}^{1/2}}$$

where X, Y, and Z are the mole fractions of the M_NBE_-M_NBE_, M_CP_-M_CP_, and M_NBE_-M_CP_ dyads in the copolymer, respectively, and Φ_NBE_ corresponds to the NBE mole fractions in the copolymer. Mean sequence lengths μ_NBE_ and μ_VNBE_ were also calculated using the following equations [53]:

(11) $${\text{μ}}_{NBE}=1+r_{NBE}\left[\frac{M_{NBE}}{M_{CP}}\right]$$

(12) $${\text{μ}}_{CP}=1+r_{CP}\left[\frac{M_{CP}}{M_{NBE}}\right]$$

The data are summarized in Table 4 (dyad monomer sequences X, Y, Z and mean sequence length). The variation of the dyad fractions with the NBE mole fraction in the copolymers is displayed in Supplementary Figure S5 of the SI. These results provide a clear picture of the copolymer structures as well as the distribution of the monomer units in the copolymer chain. It is obvious that the mole fraction of the M_CP_-M_CP_ dyads is the minority component for almost all copolymer compositions. On the contrary, the mole fraction of the M_NBE_-M_NBE_ dyads is very high for all the samples. This is direct evidence of the huge difference of the two monomer reactivity ratio values.

**Table 4:** Dyad monomer sequences X = M_NBE_-M_NBE_, Y = M_CP_-M_CP_, Z = M_NBE_-M_CP_ dyads and mean sequence lengths.

Sample	X	Y	Z	μ_NBE_	µ_CP_
20/80	0.089	0.109	0.801	1.79	1.07
40/60	0.186	0.046	0.769	2.09	1.05
50/50	0.284	0.024	0.692	2.40	1.04
60/40	0.356	0.016	0.628	2.66	1.03
20/80P	0.058	0.058	0.883	1.87	1.02
40/60P	0.249	0.009	0.741	2.42	1.01
50/50P	0.287	0.007	0.705	2.55	1.01
60/40P	0.384	0.004	0.611	2.94	1.01

#### 2.1.2. Kinetics of the Thermal Decomposition of the Statistical Copolymers

The kinetics of the thermal decomposition of the statistical copolymers was studied by TGA measurements. The activation energy, E, of mass loss upon heating was calculated using the isoconversional Ozawa-Flynn-Wall (OFW) [54,55,56] and Kissinger [57,58] methods. The OFW approach is a “model free” method, which assumes that the conversion function F(α), where α is the conversion, does not change with the alteration of the heating rate, β, for all values of α. The OFW method involves the measuring of the temperatures corresponding to fixed values of α from experiments at different heating rates β. Therefore, plotting lnβ *vs.* 1/T in the form of

(13) $$ln\text{β}=ln\frac{AE}{R}-lnF\left(a\right)-\frac{E}{RT}$$

should give straight lines with slopes directly proportional to the activation energy, where *T* is the absolute temperature, *A* is the pre-exponential factor (min^−1^), and *R* is the gas constant (8.314 J/K·mol). If the determined activation energy is the same for the various values of α, then the existence of a single-step degradation reaction can be concluded with certainty. The OFW method is the most useful method for the kinetic interpretation of thermogravimetric data, obtained from complex processes like the thermal degradation of polymers. It can be applied without the knowledge of the reaction order.

The activation energy E was also calculated from plots of the logarithm of the heating rate over the squared temperature at the maximum reaction rate, T_p_^2^, *vs.* 1/T_p_ in constant heating experiments, according to the Kissinger method using the equation:

(14) $$ln\left(\frac{\text{β}}{T_{p}^{2}}\right)=ln\frac{AE}{R}+ln\left[n{\left(1-a_{p}\right)}^{n-1}\right]-\frac{E}{RT_{p}}$$

where T_p_ and α_p_ are the absolute temperature and the conversion at the maximum weight-loss and n is the reaction order. The E values can be calculated from the slope of the plots of ln(β/T_p_^2^) versus 1/T_p_.

**Figure 2:**
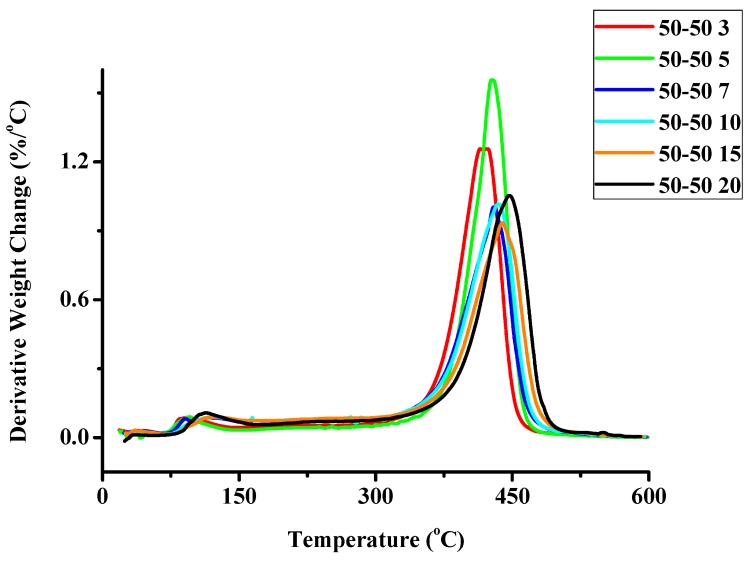
Derivative weight loss with temperature for the sample 50/50 under different heating rates.

The statistical copolymers were thermally degraded at different heating rates. The detailed results are given in the Supporting Information Section (Figures S6–S8, Tables S1–S6), whereas an example of the recorded thermograms under different heating rates is displayed in Figure 2. Differential thermogravimetry, DTG, showed that a single decomposition peak appears for both homopolymers. The thermal decomposition of PCP starts and is completed at lower temperatures than that of PNBE. However, the temperature at the maximum rate of decomposition of PCP is higher than that of PNBE. These results imply that the mechanism of decomposition is different between the two homopolymers reflecting the differences in their chemical structure. DTG revealed that a single decomposition peak appears for the statistical copolymers as well. The temperature at the maximum rate of their decomposition lies between the values of the respective homopolymers.

**Figure 3:**
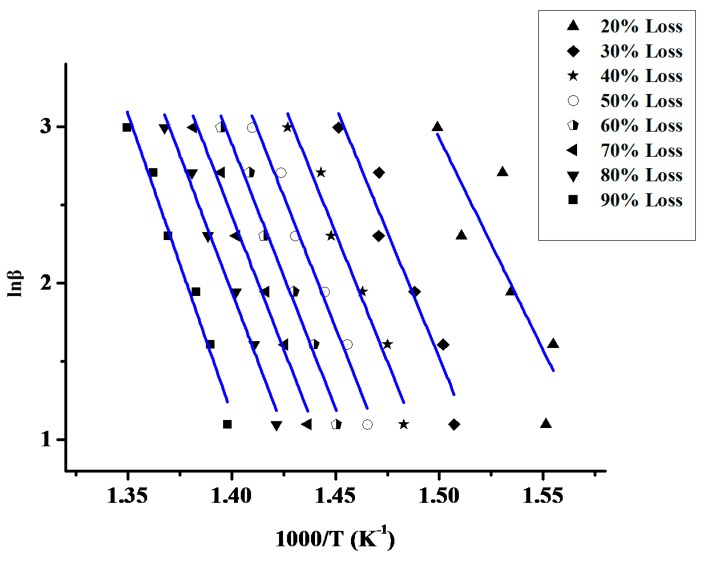
Ozawa-Flynn-Wall plots lnβ *vs.* 1/T for the sample 20/80 at different heating rates.

The linearity of the OFW diagrams in a broad range of conversions proves that it is an efficient method for the analysis of the thermal decomposition of the copolymers. A characteristic example is given in Figure 3. More examples are incorporated in the SI Section (Supplementary Figures S9 and S10). However, deviations from the linearity were observed in very low or very high conversions, since these results refer to the decomposition of parts of the macromolecules that are not representative of the overall copolymeric chains, due to the statistical character of the distribution of the different monomer units.

The activation energy values of the PCP homopolymer decrease upon increasing the decomposition conversion, whereas the opposite behavior was obtained for the PNBE homopolymer due to the different decomposition mechanism of the two samples. The activation energy values of the copolymers change considerably upon changing the decomposition conversion, indicating that the decomposition mechanism is complex during the thermal decomposition. This behavior resembles that of the PNBE homopolymer, due to the very high content in NBE units along the copolymer chains, which reflects the much higher reactivity ratio of NBE, compared to that of CP. The data from the OFW diagrams are provided in Table 5. The observed relatively-good agreement of the Ea values obtained between the OFW and the Kissinger methods can be attributed to the same effect. The Ea values obtained from the Kissinger method are given in Table 6, whereas characteristic plots are provided in Figures S11–S14 in the SI.

**Table 5:** Activation energies for the homopolymers and the statistical copolymers from the OFW method for different degrees of decomposition.

Weight Loss	PCP	PNBE	80/20	60/40	50/50	40/60	20/80	20/80P	40/60P	50/50P	60/40P	80/20P
10	340.46	-	36.681	105.75	-	174.59	-	191.26	105.10	-	-	-
20	326.49	-	64.583	157.38	-	201.36	225.14	239.50	188.00	188.20	-	-
30	302.21	-	117.89	252.66	17.351	273.86	267.63	243.78	211.01	210.91	176.30	176.66
40	289.24	296.81	164.37	269.96	249.50	290.49	274.11	255.06	226.03	225.70	218.32	225.88
50	275.61	377.79	206.77	273.53	328.57	251.33	279.77	253.71	233.46	233.52	242.20	231.98
60	266.88	332.89	253.41	268.46	336.38	205.11	281.26	254.62	238.20	238.22	251.72	262.36
70	260.73	308.28	316.18	267.63	320.26	43.964	284.09	254.25	239.55	235.10	255.00	267.26
80	257.65	-	287.08	300.30	283.09	32.316	290.99	253.87	242.52	242.60	254.75	274.41
90	255.90	-	225.23	-	243.77	-	318.92	256.23	250.26	250.29	187.75	267.26

**Table 6:** Activation energies for the homopolymers and the statistical copolymers from the Kissinger method.

Sample	Ea
PCP	227.24
PNBE	247.11
20/80	190.30
40/60	214.21
50/50	316.54
60/40	214.49
80/20	275.64
20/80P	253.69
40/60P	249.55
50/50P	243.62
60/40P	236.62
80/20P	259.13

### 2.2. Statistical Copolymers of NBE with CP in the Presence of Triphenylphosphine

The rapid propagation of most typical monomers in ROMP, employing Ru catalysts usually leads to the formation of polymers having rather broad molecular weight distributions. However, Bielawski and Grubbs showed that the addition of bulky phosphines in the polymerization media retards the propagation reaction [33]. The catalyst contains two PCy_3_ ligands, one of which reversibly dissociates from the metal center in solution to yield the active ROMP site [32]. By adding excess phosphine to the solution, the binding equilibrium between Ru and phosphine ligand is shifted towards the inactive bound state, thereby slowing propagation without significantly slowing initiation, and thus producing a better-controlled polymerization system. It was found that in order to have better initiation characteristics and therefore, better control over the molecular characteristics of the produced polymers it is better to use phosphines that are relatively more labile compared to PCy_3_. It was found that k_i_/k_p_, is equal to 10.2 when the polymerization takes place in the presence of excess PPh_3_ (5 fold molar excess over the catalyst), whereas k_i_/k_p_ equals 1.02 in the presence of excess PCy_3_ (5 fold molar excess over the catalyst) [33]. Therefore, a similar approach was applied for the copolymerization of NBE with CP to investigate the effect of PPh_3_ in the monomer reactivity ratios. A second series of ROMP copolymerization reactions was conducted by adding an excess of PPh_3_ (molar ratio [PPh_3_]/[catalyst] = 4/1) in the Grubbs catalyst solution. In all cases the conversion was kept relatively low to satisfy the differential copolymerization equation. The reactions were accomplished at room temperature in CH_2_Cl_2_ solutions. This is a distinct difference with the previous system, where the copolymerization was conducted in the absence of excess phosphine at 0 °C. Our purpose was to perform the copolymerization reaction under conditions that will allow the incorporation of a substantial amount of CP units along the copolymeric chains. In the absence of excess phosphine the polymerization of NBE is very fast. In order to slow down the reaction and achieve sufficient incorporation of CP units the copolymerization was conducted at 0 °C. However, in the presence of excess phosphine when the copolymerization was conducted at 0 °C the product was almost pure PNBE homopolymer with negligible incorporation of CP units along the polymeric chain. This result clearly shows that the decrease of the polymerization rate of CP in the presence of PPh_3_ is much more pronounced than that of NBE at this temperature. Therefore, in order to achieve sufficient incorporation of CP units during the copolymerization the reaction was allowed to take place at room temperature [29,59]. The molecular characteristics of the samples are given in Table 1. The samples are symbolized as previously mentioned but with the addition of the letter P. Therefore, sample 40/60P indicates the copolymer for the synthesis of which 40% NBE and 60% CP was employed as the molar feed composition in the presence of PPh_3_. The molecular weights were measured by SEC and NMR spectroscopy was employed to calculate the composition of these copolymers by integrating the same peaks, as previously reported. It is obvious that products of narrower molecular weight distribution were obtained when the copolymerization is conducted in the presence of excess phosphine. The SEC traces of the copolymers and a characteristic ^1^H-NMR spectrum are given in the SI (Supplementary Figures S15 and S16).

#### 2.2.1. Monomer Reactivity Ratios and Statistical Analysis of the Copolymers

**Figure 4:**
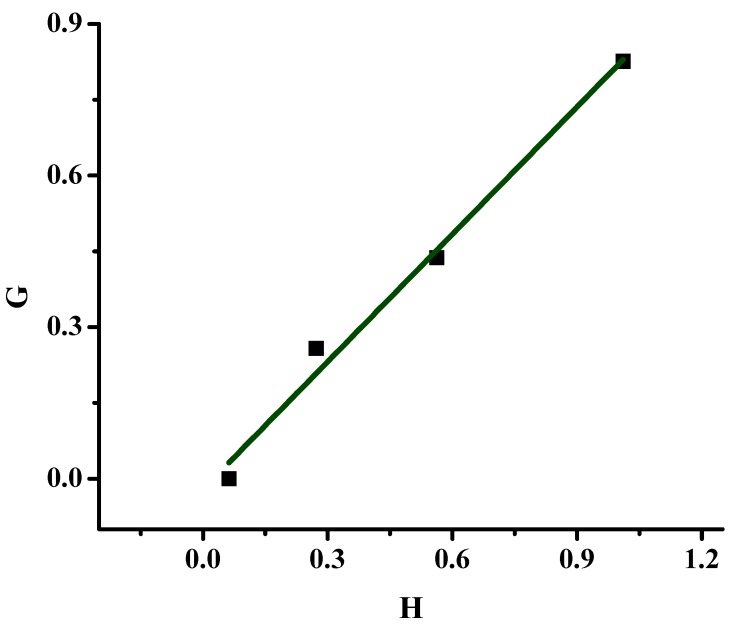
FR plot of the statistical copolymers prepared in the presence of PPh_3_.

The monomer reactivity ratios were calculated using the Finemann-Ross (FR), inverted Finemann-Ross (IFR), and Kelen-Tüdos (KT) graphical methods along with the computer program COPOINT in a similar way as previously reported. The copolymerization data for all systems are provided in Table 2. The FR graphical plot is given in Figure 4, whereas the graphical plots of the other methods are given in the SI (Figures S17 and S18) and the reactivity ratios in Table 3. As previously mentioned, the sample 80/20P was not taken into account for the calculation of the reactivity ratios, since the conversion of the polymerization reaction was much higher than that of the other samples, thus deviating from the copolymerization equation.

All methods provide similar data concerning the reactivity ratios for both monomers. According to the data obtained by the Kelen-Tüdos method r_NBE_ = 0.87 and r_CP_ = 0.02. These results imply that in this case as well a non-ideal, non-azeotropic copolymerization takes place with an increased rate of NBE incorporation into the copolymer structure compared to that of CP incorporation. It is characteristic that in the presence of PPh_3_ the r_NBE_ was further increased, whereas the r_CP_ value was decreased. This result indicates that the effect of PPh_3_ in reducing the rate of propagation is much more pronounced in the case of CP polymerization. Finally, the copolymeric chains are even richer in NBE units when the copolymerization is conducted in the presence of PPh_3_. This behavior is also depicted in the dyad monomer sequences X, Y, and Z, which are displayed in Table 4 and in the SI (Supplementary Figure S19).

#### 2.2.2. Kinetics of the Thermal Decomposition of the Statistical Copolymers

**Figure 5:**
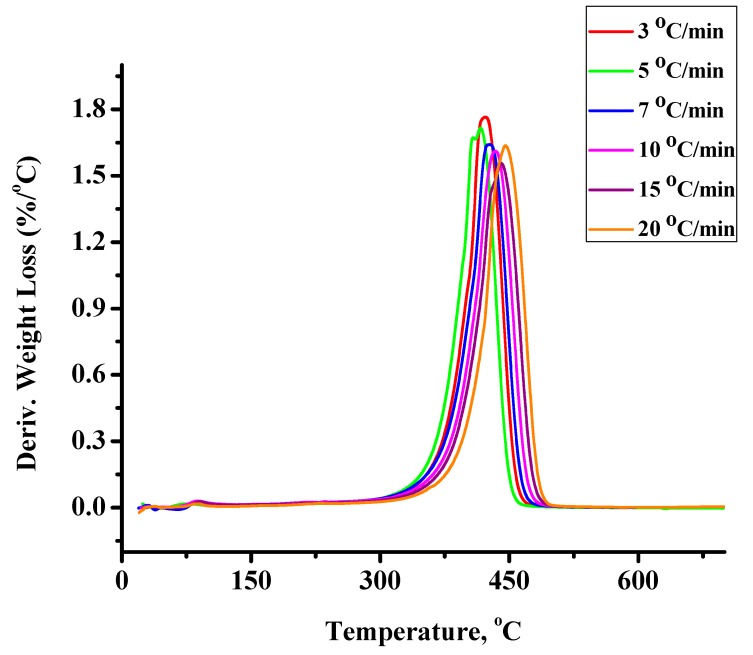
Derivative weight loss with temperature for the sample 20/80P under different heating rates.

The thermal decomposition of the statistical copolymers synthesized in the presence of PPh_3_ was also studied by TGA measurements. The results are given in the SI (Supplementary Figures S20–S22 and Tables S7–S12), whereas an example of the recorded thermograms under different heating rates is displayed in Figure 5. Differential thermogravimetry, DTG, showed that, also in this case, single decomposition peaks appeared for all copolymers. The activation energy, Eα, of mass loss upon heating was calculated using the OFW and Kissinger methods and the corresponding diagrams of these methods are given in the SI (Supplementary Figures S23–S27). The activation energies for the homopolymers and the statistical copolymers calculated by the two different methods are presented in Table 5 and Table 6. The temperature range of the thermal decomposition of the copolymers prepared in the presence of PPh_3_ is narrower than in the case of the copolymers prepared without PPh_3_. This is attributed to the fact that in the former case the copolymers are more uniform, since they are richer in NBE units and, thus, resemble more to the PNBE homopolymers.

The Ea values measured by the OFW method do not vary appreciably with conversion indicating that the decomposition mechanism is rather simple. This is further supported by the existence of a uniform polymeric structure. In addition, the variation of the Ea values between the different copolymers is very small, due to the high NBE content for all samples. The results for the OFW and the Kissinger methodologies are in very good agreement fortifying the conclusion that the decomposition mechanism is rather simple.

In conclusion, the presence of PPh_3_ during the copolymerization considerably affects the thermal decomposition of the produced copolymers. The change of the reactivity ratios leads to the formation of copolymers with fewer CP units and a more random placement compared to the samples prepared in the absence of PPh_3_. Therefore, the decomposition mechanism is simpler and resembles that of PNBE homopolymers.

## 3. Experimental Section

### 3.1. Materials

Cyclopentene (96% Aldrich, St. Louis, MO, USA) was dried by stirring over calcium hydride and was distilled under vacuum before use. Dichloromethane was purified over calcium hydride overnight, degassed by freeze-pump-thaw cycles and vacuum distilled. Norbornene (99% Aldrich) was dissolved in dichloromethane and was vacuum distilled from calcium hydride. Bis(tricycloshexylphosphine)benzylidene ruthenium(IV) dichloride (a Grubbs “first-generation” Ru initiator), PPh_3_ and all other reagents were used as received from Aldrich.

### 3.2. Copolymerization Studies

In order to study the copolymerization of NBE with CP, a set of five experiments was conducted. Different feed ratios were employed each time (monomer molar ratios: 80/20, 60/40, 50/50, 40/60, and 20/80), and the copolymerization reactions were quenched at low (<20%) yields. The copolymerization procedure was monitored by size exclusion chromatography, SEC. The experimental results were processed on the basis of the Finemann-Ross (FR), Inverted Finemann-Ross (IFR), and Kelen-Tüdos (KT) equations. The computer program COPOINT was also employed.

### 3.3. Copolymerization Reactions

Two different series of NBE-CP copolymerization reactions were conducted. In the first, statistical NBE-CP copolymerizations at five different molar feed monomer compositions were carried out under argon atmosphere at 0 °C. In a typical procedure, the Grubbs’ catalyst (20 mg) was dissolved in dichloromethane (60 mL), distilled from CaH_2_. A mixture of CP and NBE (total amount of ~3.6 × 10^−3^ mol or total concentration of ~6 × 10^−5^ mol/mL), both dried by distillation from CaH_2_, was then added to the reaction mixture and the copolymerization was terminated by adding a small amount of ethyl vinyl ether, in order to keep the yield below 20%. The polymer was dissolved in dichloromethane and purified using a silica-gel column to remove the remaining catalyst. Finally, it was precipitated into a large excess of methanol and dried under vacuum. In the second series of copolymerization reactions, the NBE-CP copolymers were synthesized by adding an excess of triphenylphosphine (25 mg) to the catalyst solution. The copolymerizations, for each feed monomer composition, were conducted in a similar way, as previously reported, but were carried out at room temperature.

### 3.4. Characterization Techniques

Size exclusion chromatography (SEC) experiments were carried out using a modular instrument consisting of a Waters Model 510 pump, a Waters Model U6K sample injector, a Waters Model 401 differential refractometer (Milford, MA, USA), and a set of 4 μ-Styragel columns with a continuous porosity range from 10^6^ to 10^3^ Å. The columns were housed in an oven thermostatted at 40 °C. THF or CHCl_3_ were the carrier solvents at a flow rate of 1 mL/min. The instrument was calibrated with polystyrene standards.

^1^H-NMR spectra were recorded in chloroform-*d* at 30 °C with a Varian Unity Plus 300/54 NMR spectrometer (Palo Alto, CA, USA).

The kinetics of the thermal decomposition of the copolymers was studied by thermogravimetric analysis (TGA) employing a Q50 TGA model from TA instruments. Samples were placed in platinum crucibles. An empty platinum crucible was used as reference. The samples were heated from ambient temperatures to 600 °C in a 60 mL/min flow of N_2_ at heating rates of 1, 3, 5, 7, 10, 15, and 20 °C/min.

## 4. Conclusions

Statistical copolymers of NBE and CP were synthesized in a controlled manner via ROMP using the 1st-generation Grubbs’ catalyst in the presence and absence of triphenylphosphine. In the presence of phosphine the copolymerizations were conducted at room temperature and at 0 °C in the absence of phosphine. The reactivity ratios of the different monomers were estimated using linear graphical methods. The r_NBE_ values were considerably higher than the corresponding r_CP_ values in all cases, meaning that a kinetic preference exists for the incorporation of NBE in the copolymer structure. These results were confirmed by the calculation of the monomer dyad sequence fractions. The use of an excess of phoshine during the copolymerization leads to lower polydispersities. PPh_3_ has a remarkable effect as it acts as a polymerization regulator by shifting the metal-ligand binding equilibrium. Finally, the kinetics of the thermal decomposition of the copolymers was studied by thermogravimetric analysis, TGA, in the frame of the Ozawa-Flynn-Wall and Kissinger methods. The thermal stability of both homopolymers is similar, although slightly higher decomposition temperatures and broader decomposition temperature ranges were obtained for PNBE. The mechanism of the thermal decomposition is rather complex for the statistical copolymers prepared in the absence of PPh_3_. However, in the presence of phosphine the lower incorporation of CP units along the copolymeric chains leads to a more simple decomposition mechanism, resembling that of PNBE homopolymers.

## Supplementary Materials

Supplementary materials can be accessed at: http://www.mdpi.com/1420-3049/20/09/15597/s1.
