# Supplementary material for: Statistical Ring Opening Metathesis Copolymerization of Norbornene and Cyclopentene by Grubbs’ 1st-Generation Catalyst
Source: Molecules. 2015 Aug 27;20(9):15597–615. doi: 10.3390/molecules200915597 (PMC6331872; doi:10.3390/molecules200915597)
Supplement: Supplementary file 1 [file molecules-20-15597-s001.pdf]

Supporting Information

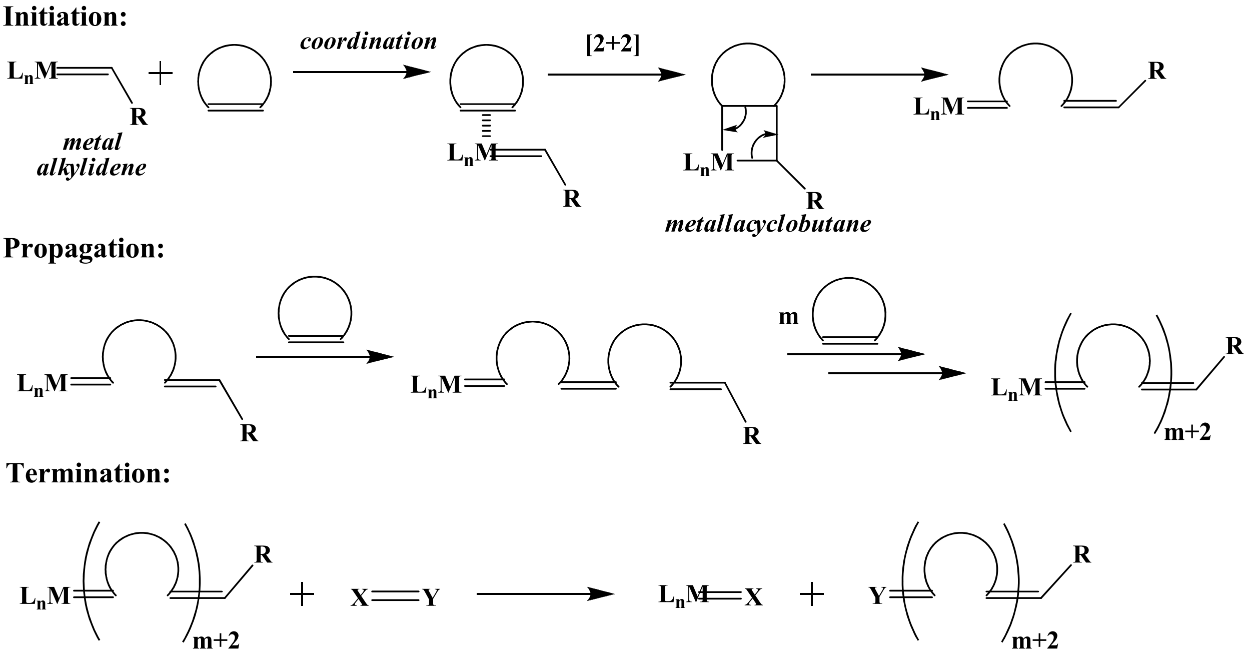

Scheme S1. General mechanism to a typical ROMP reaction.

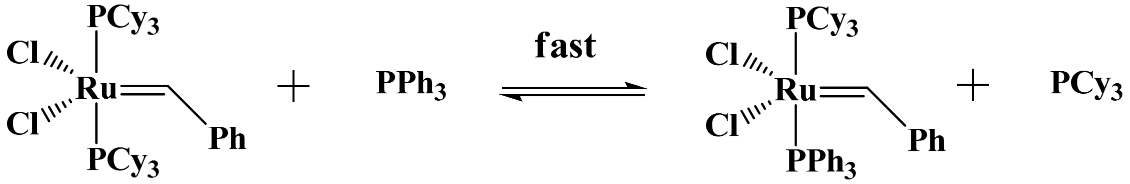

Scheme S2. Phosphine exchange reaction.

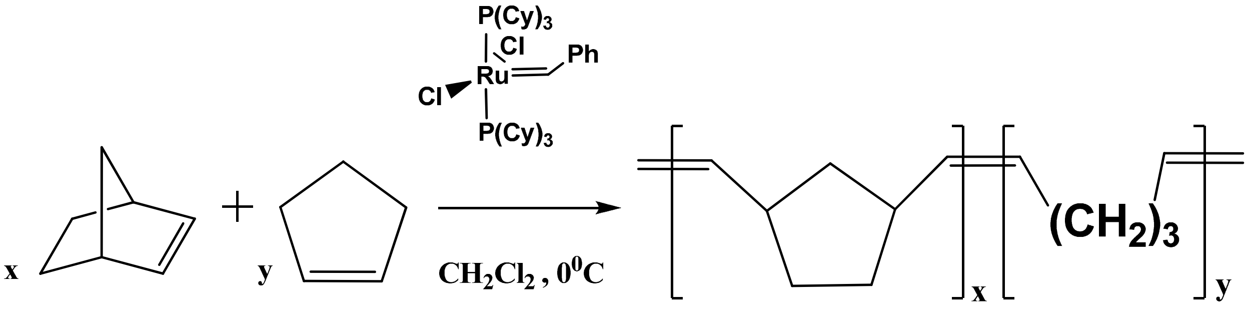

Scheme S3. Synthesis of the statistical copolymers P(NBE-*co*-CP).

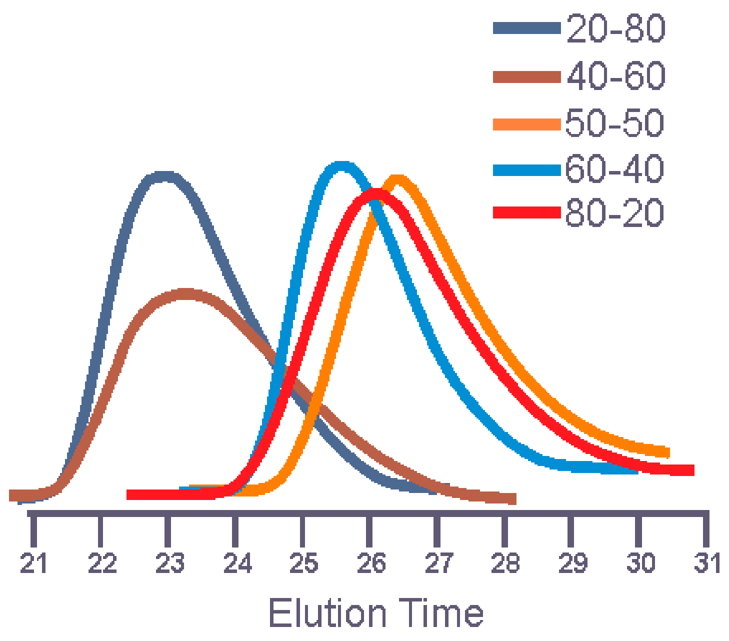

Figure S1. SEC chromatograms of the statistical copolymers PNBE-*co*-PCP in THF at 40 °C.

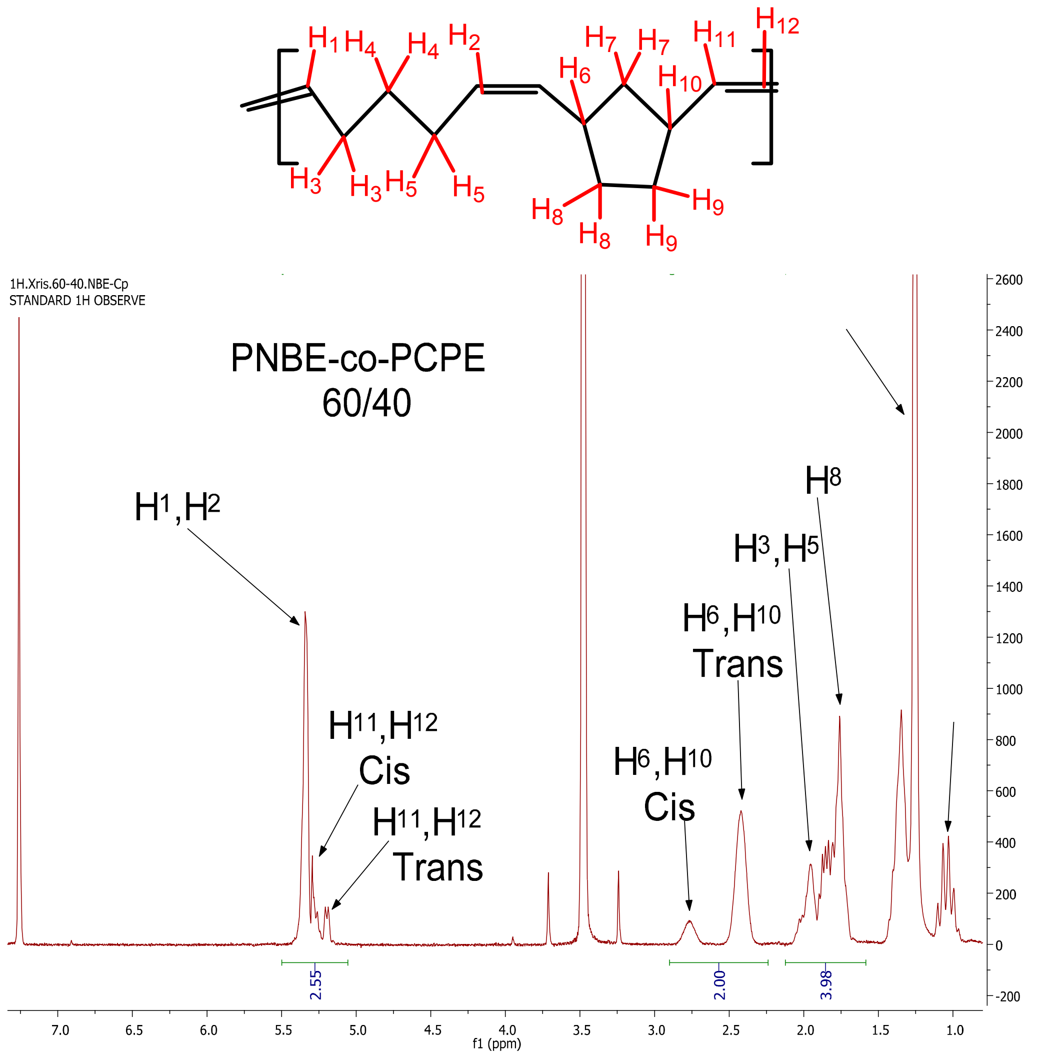

Figure S2. ^{1}H-NMR spectrum of sample PNBE-*co*-PCP 60/40 in $CDCl_3$ at 25 °C.

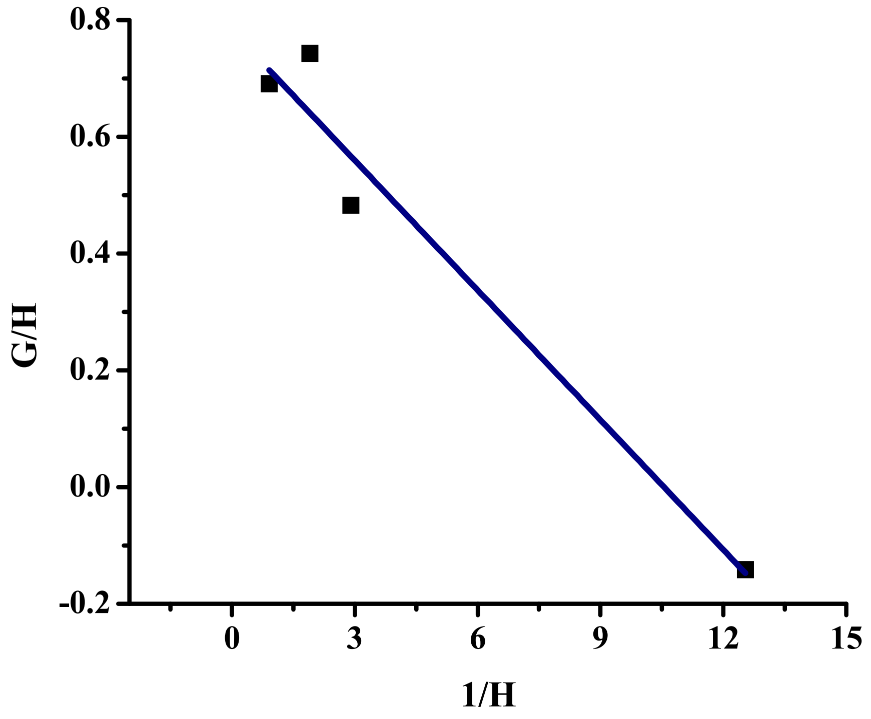

Figure S3. i-FR plot of the statistical copolymers.

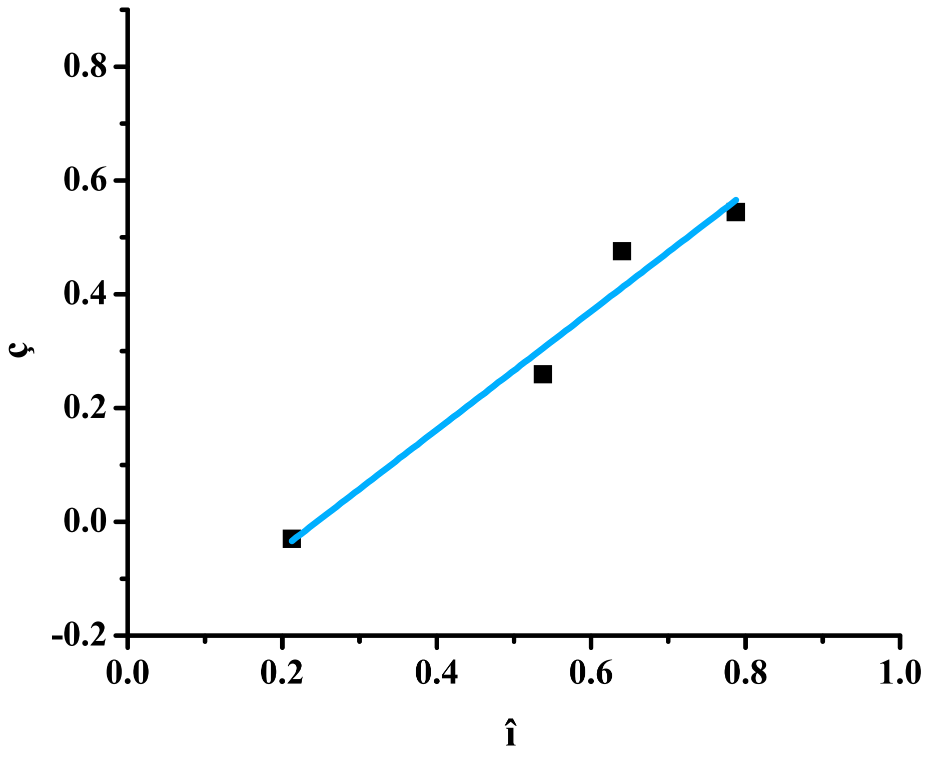

Figure S4. KT plot of the statistical copolymers.

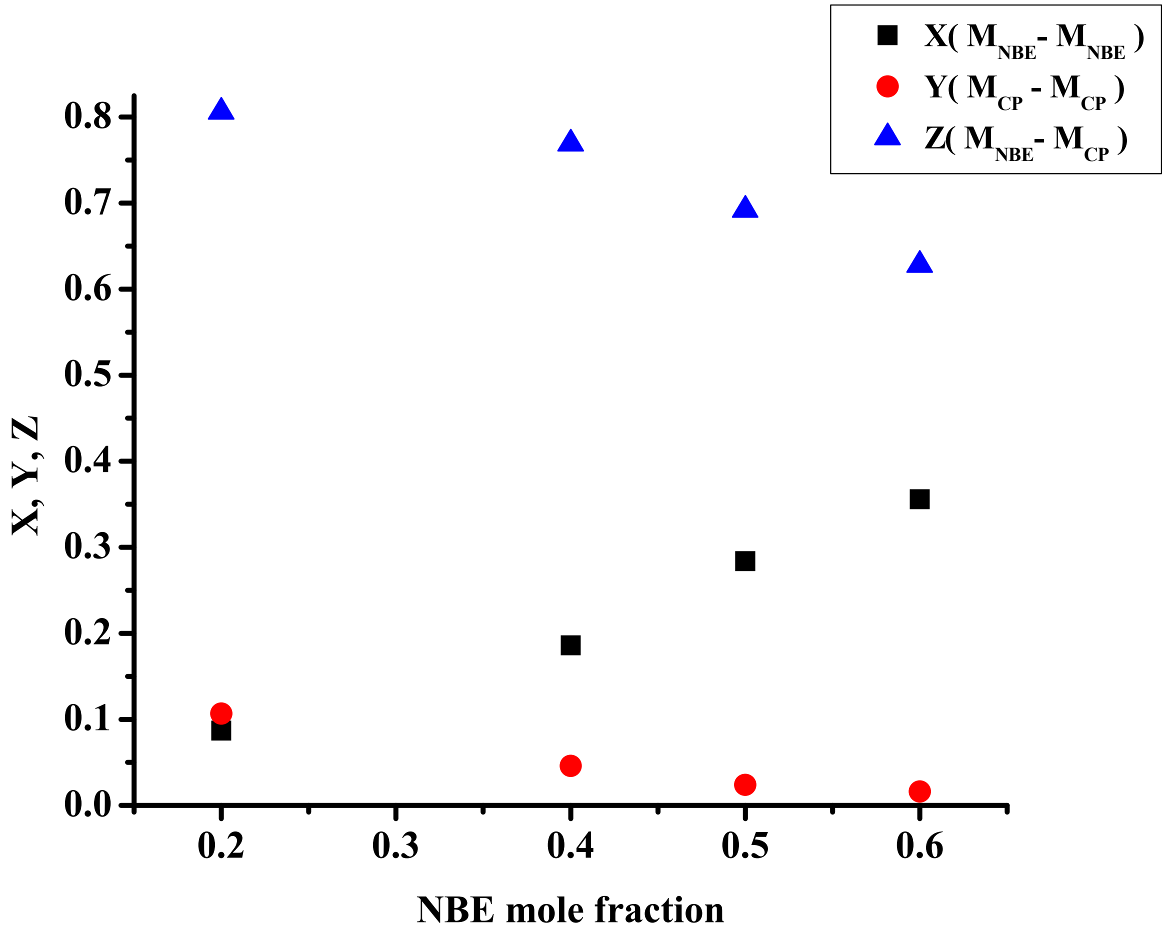

Figure S5. Dyad monomer sequence fractions *vs.* the NBE mole fraction for the statistical copolymers: X = MNBE-MNBE , Y = MCP-MCP, Z = MNBE-MCP dyads.

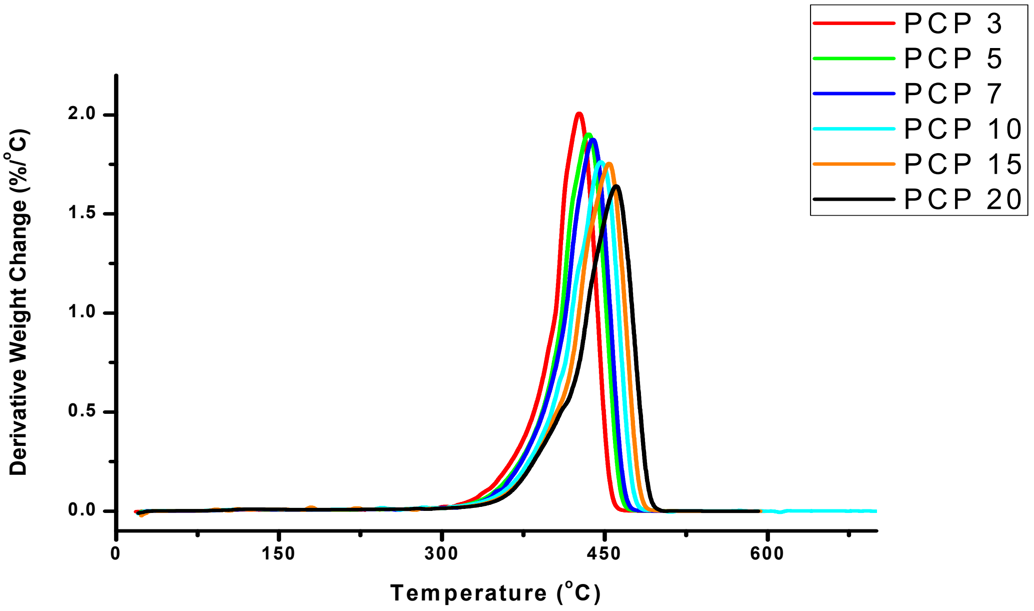

Figure S6. Derivative weight loss with temperature for the sample PCP under different heating rates.

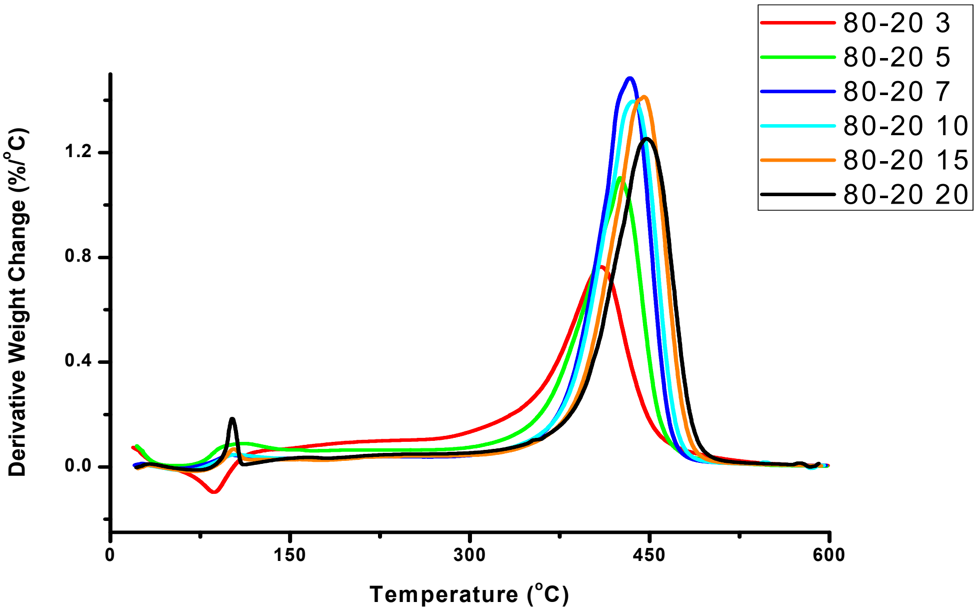

Figure S7. Derivative weight loss with temperature for the sample PNBE-*co*-PCP 80/20 under different heating rates.

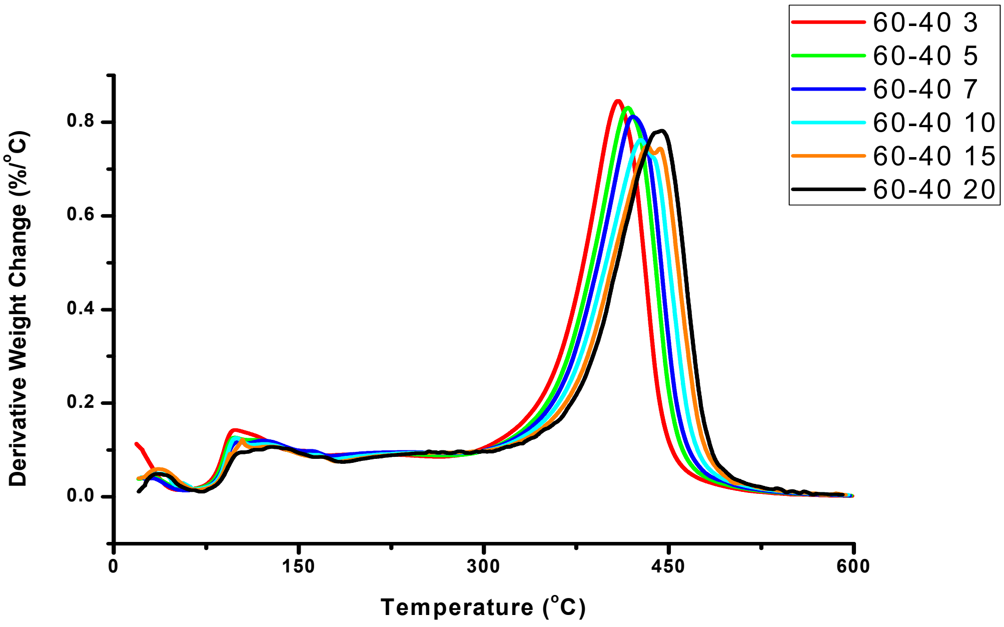

Figure S8. Derivative weight loss with temperature for the sample PNBE-*co*-PCP 80/20 under different heating rates.

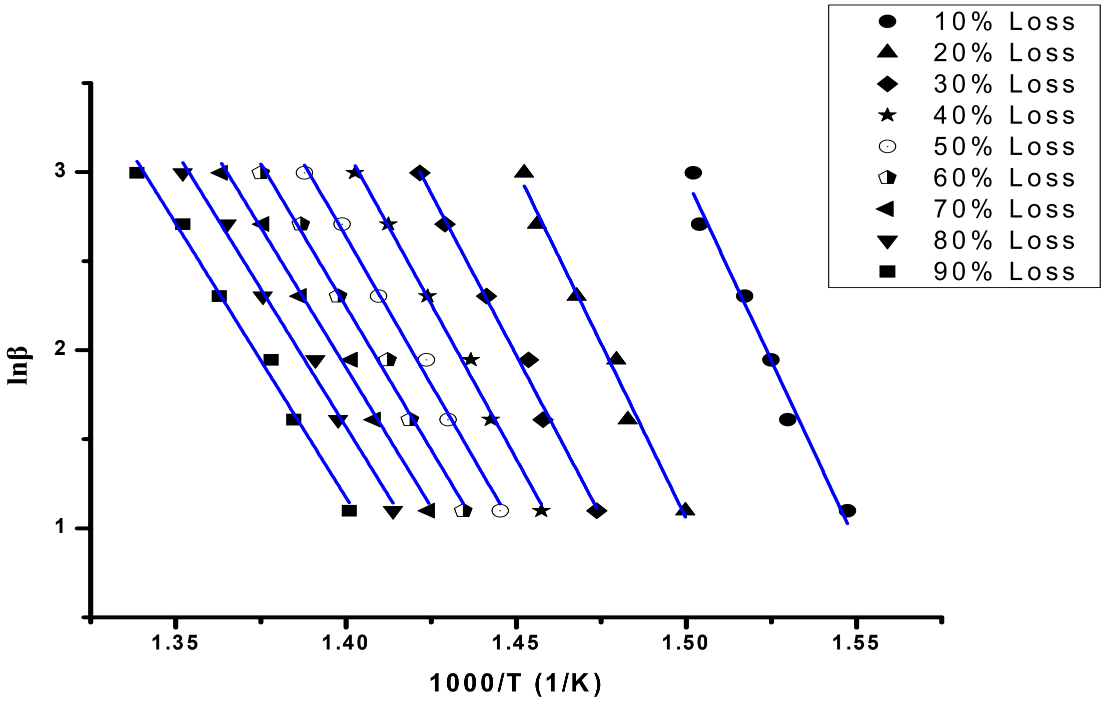

Figure S9. Ozawa-Flynn-Wall plots lnβ *vs.* 1/T for the sample PCP at different heating rates.

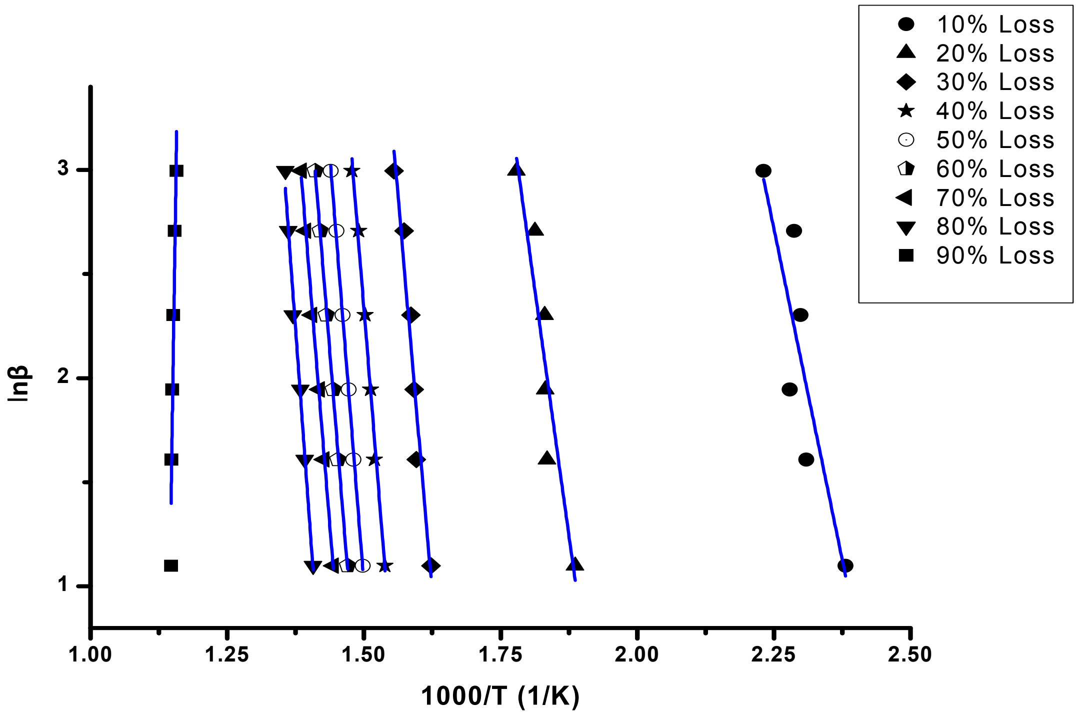

Figure S10. Ozawa-Flynn-Wall plots lnβ *vs.* 1/T for the sample 60/40 at different heating rates.

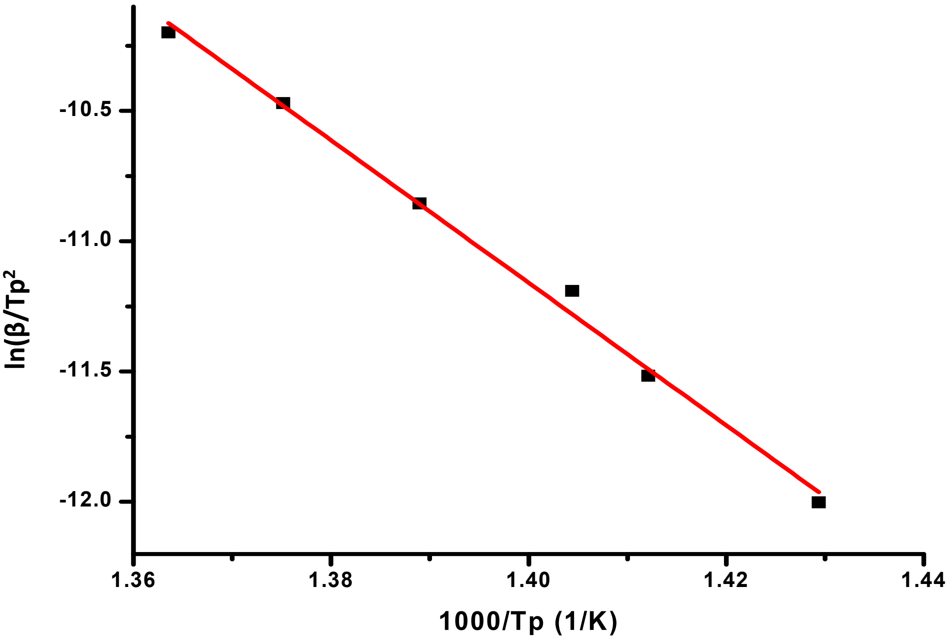

Figure S11. Kissinger plot ln(β/T_p^2) *vs.* 1000/T_p for sample PCP.

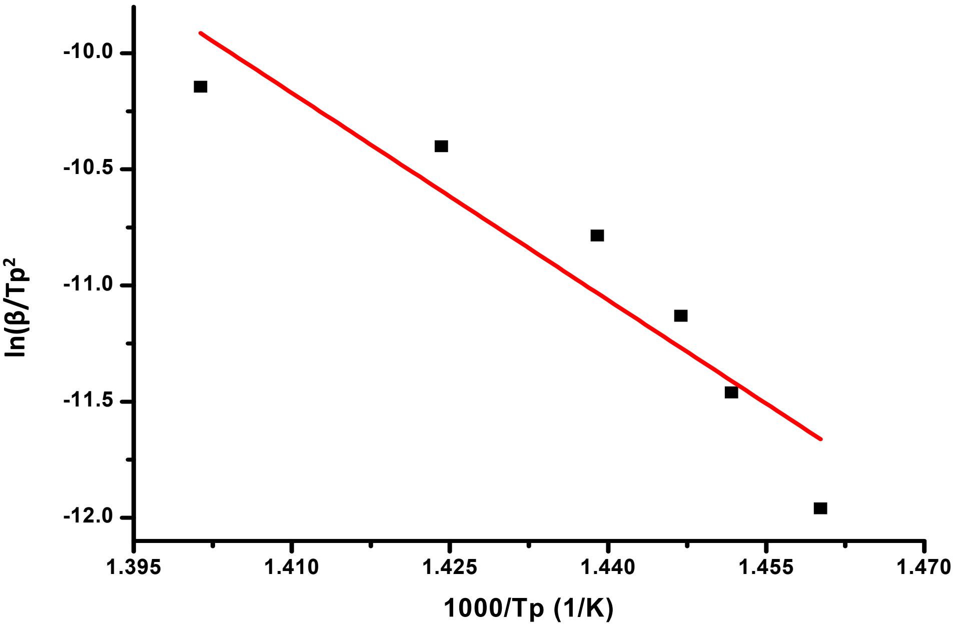

Figure S12. Kissinger plot ln(β/T_p^2) *vs.* 1000/T_p for sample PNBE.

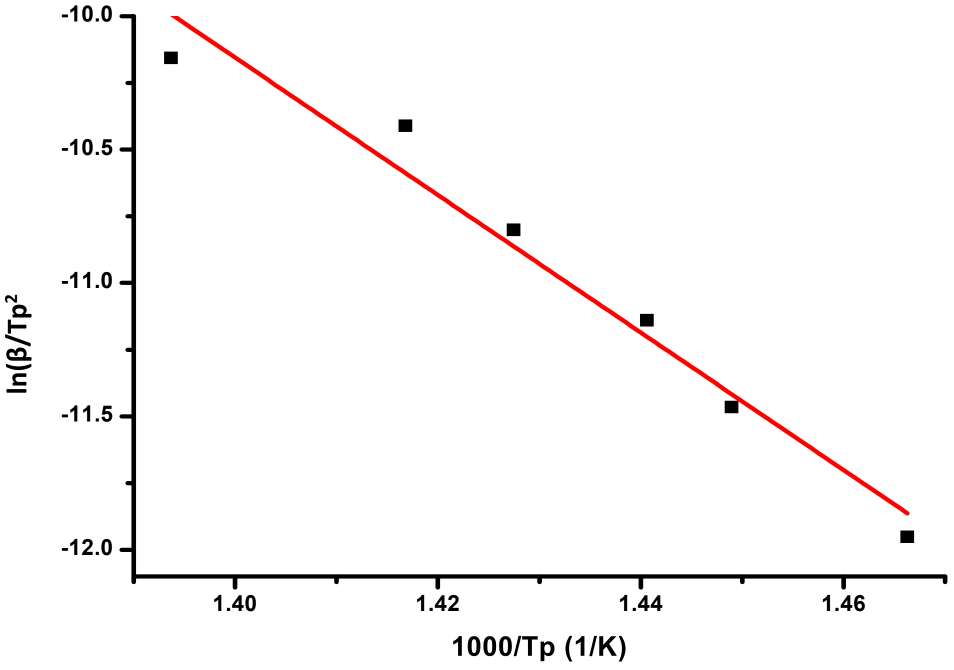

Figure S13. Kissinger plot $\ln(\beta/T_p^2)$ *vs.* $1000/T_p$ for sample 60/40.

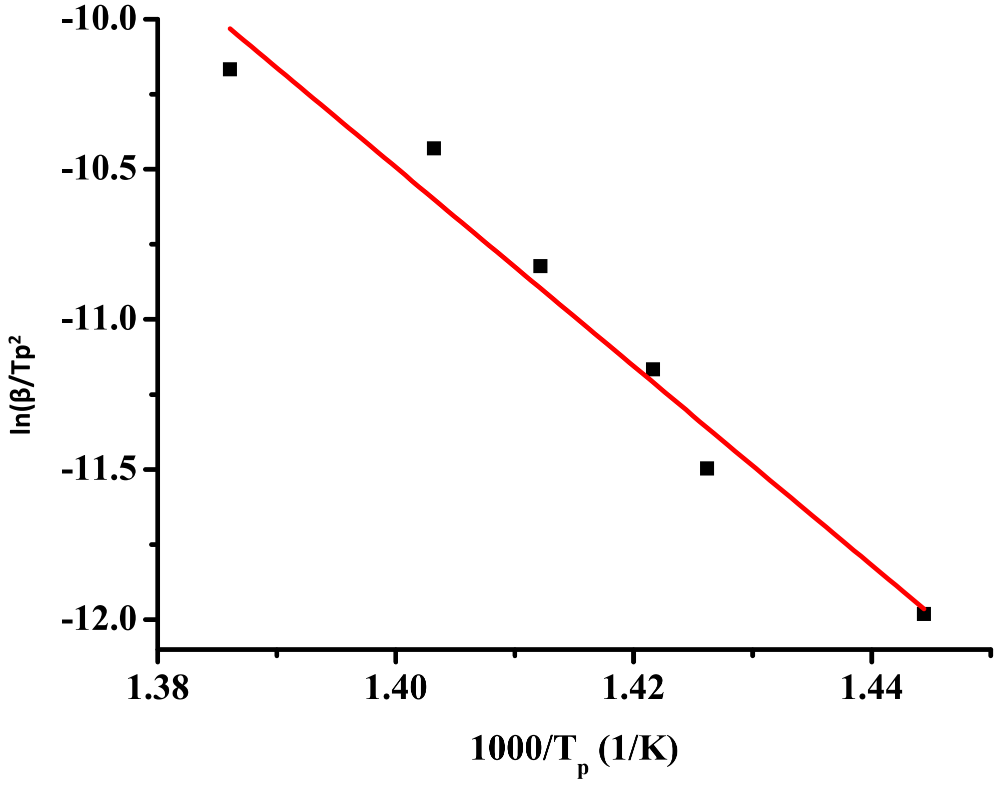

Figure S14. Kissinger plot $\ln(\beta/T_p^2)$ *vs.* $1000/T_p$ for sample 20/80.

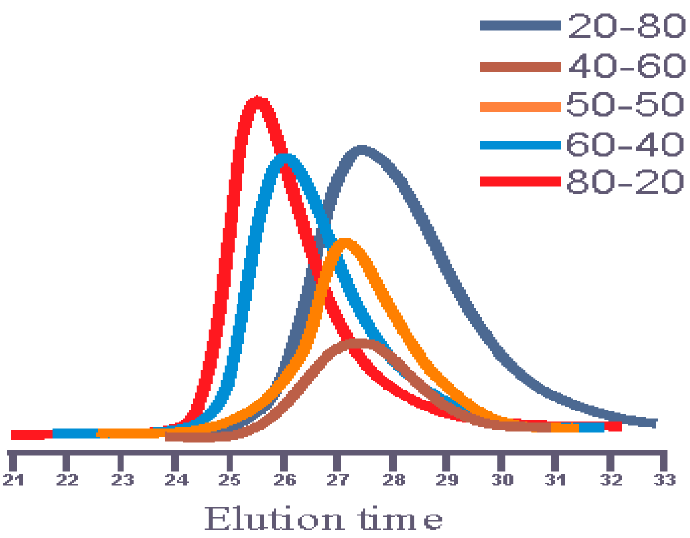

Figure S15. SEC chromatograms of the statistical copolymers PNBE-*co*-PCP in the presence of PPh_3 in THF at 40 °C.

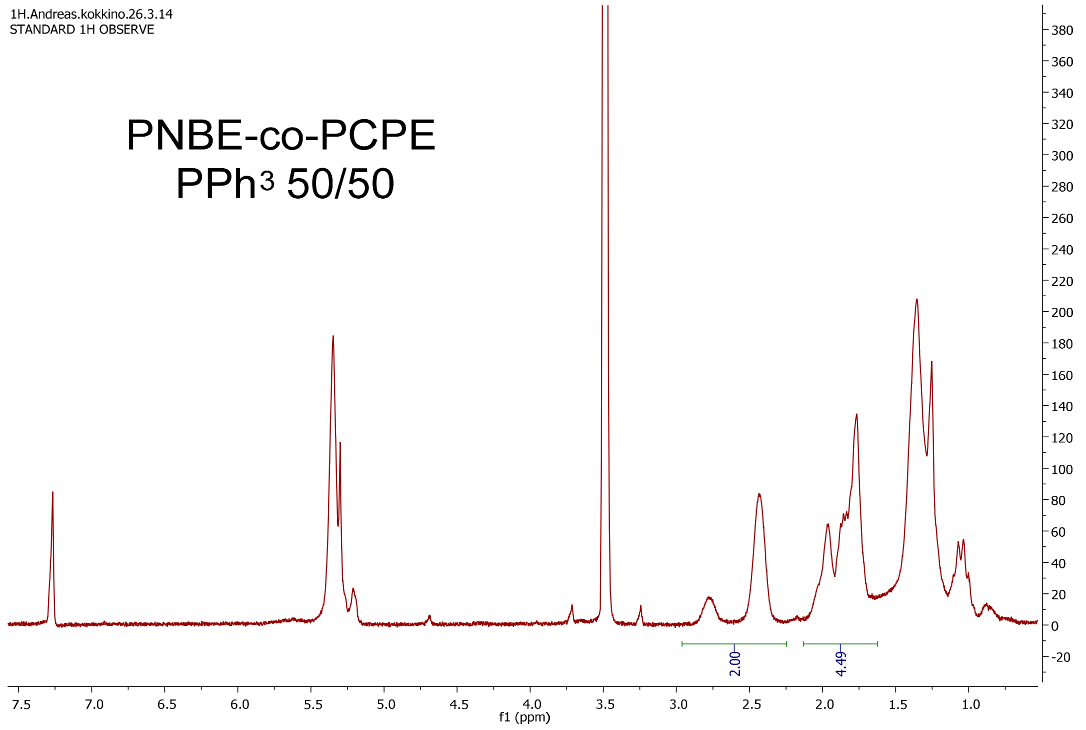

Figure S16. ^{1}H-NMR spectrum of sample PNBE-*co*-PCP 50/50 in the presence of PPh_3 in $CDCl_3$ at 25 °C.

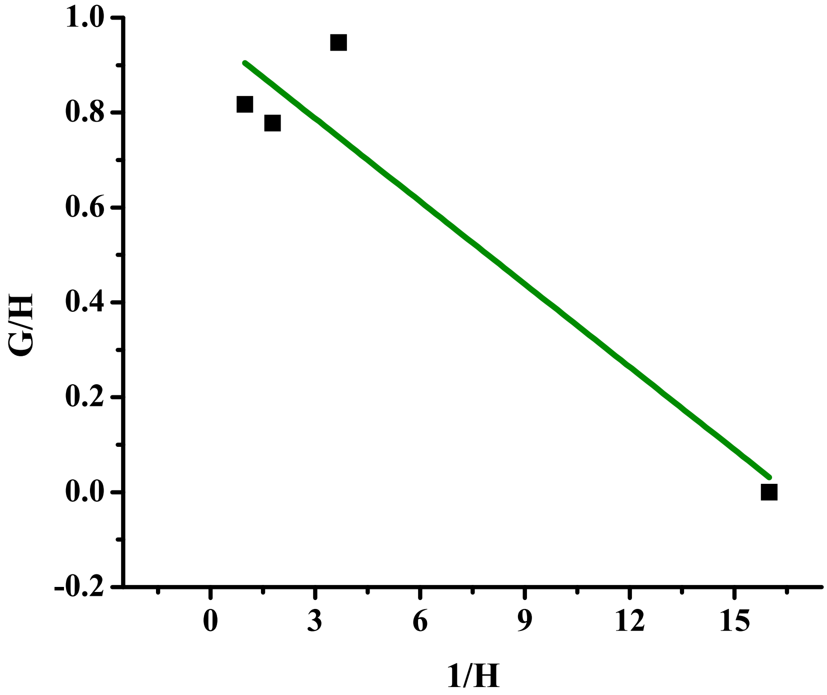

Figure S17. i-FR plot of the statistical copolymers prepared in the presence of PPh_3.

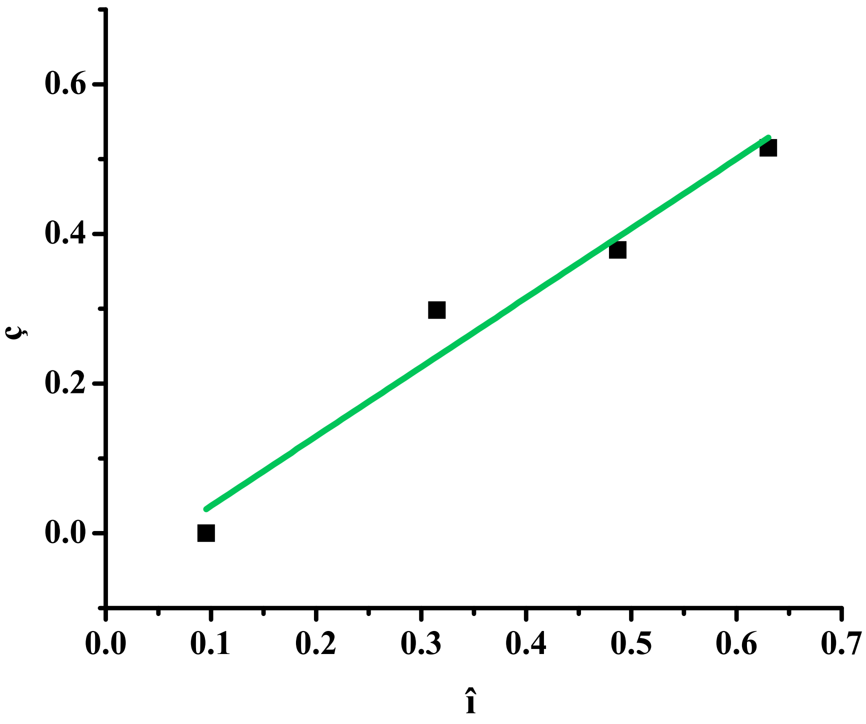

Figure S18. KT plot of the statistical copolymers prepared in the presence of PPh_3.

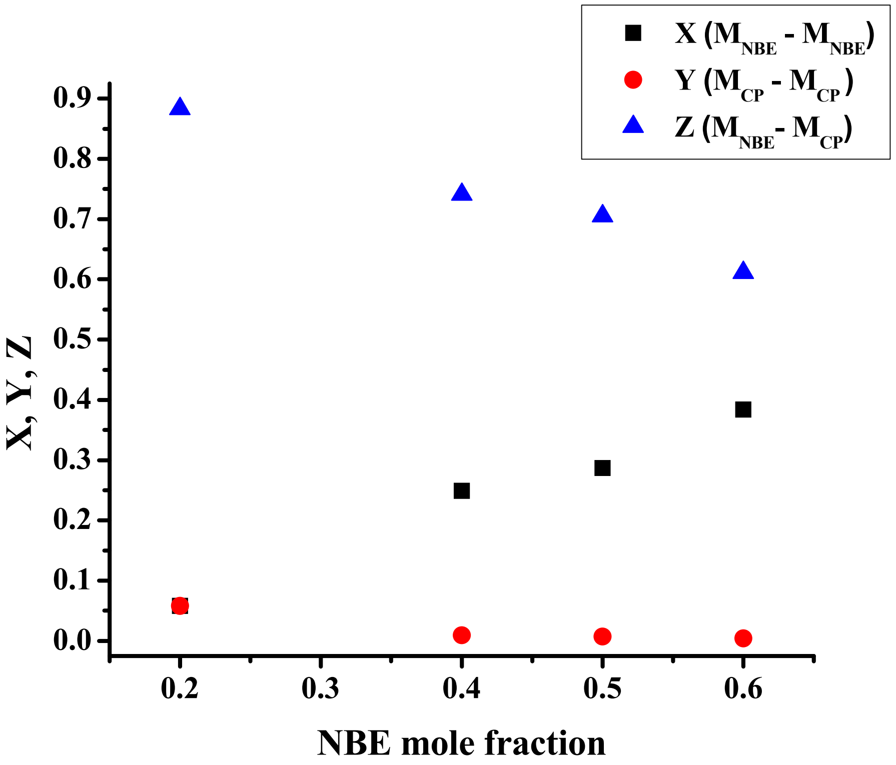

Figure S19. Dyad monomer sequence fractions *vs.* the NBE mole fraction for the statistical copolymers prepared in the presence of PPh_3: X = M_{NBE}-M_{NBE}, Y = M_{CP}-M_{CP}, Z = M_{NBE}-M_{CP} dyads.

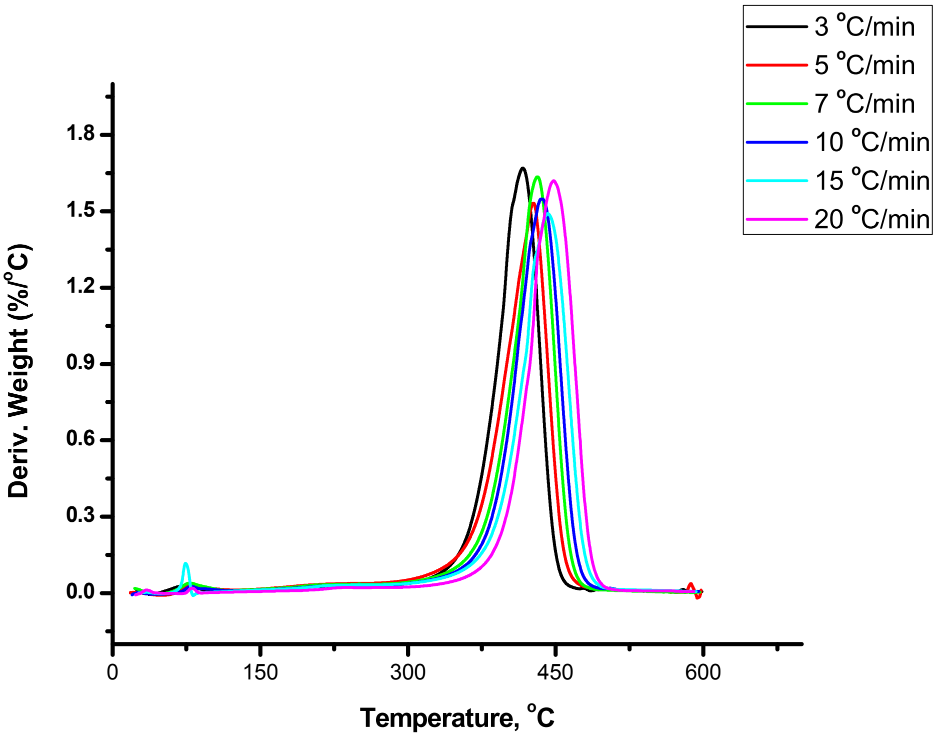

Figure S20. Derivative weight loss with temperature for the sample PNBE-*co*-PCP 40/60P under different heating rates.

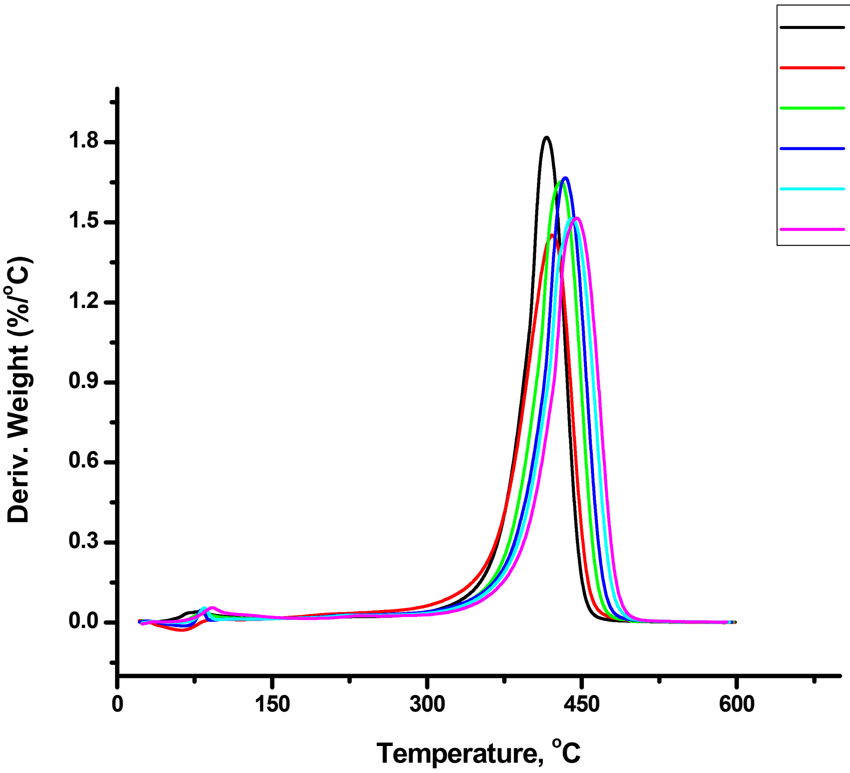

Figure S21. Derivative weight loss with temperature for the sample PNBE-*co*-PCP 50/50P under different heating rates.

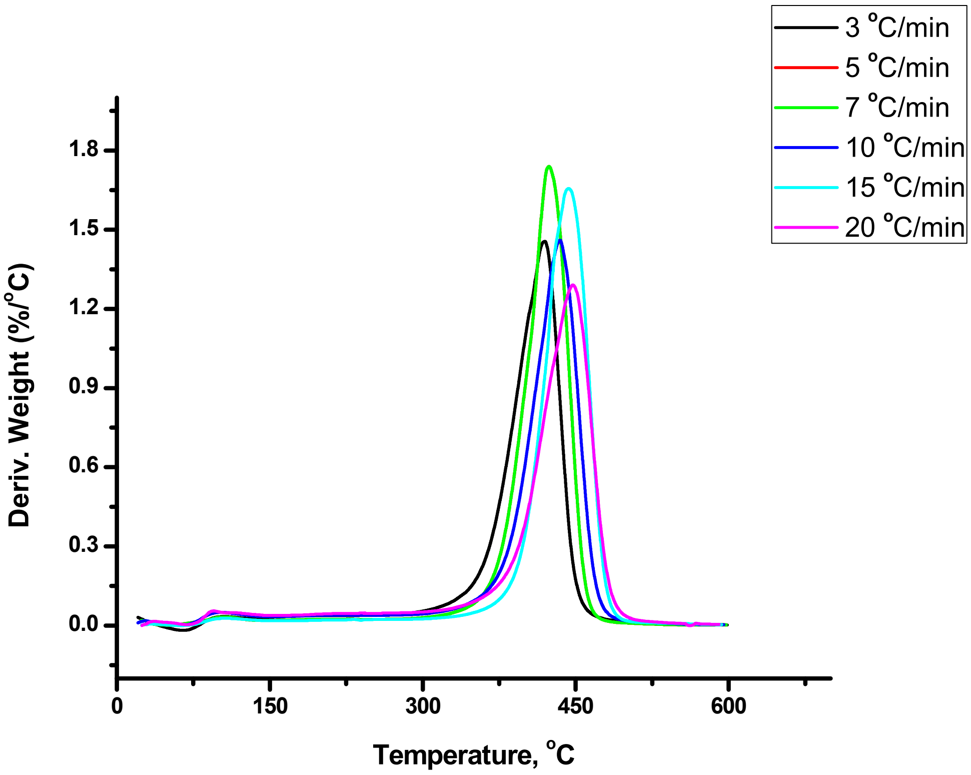

Figure S22. Derivative weight loss with temperature for the sample PNBE-*co*-PCP 80/20P under different heating rates.

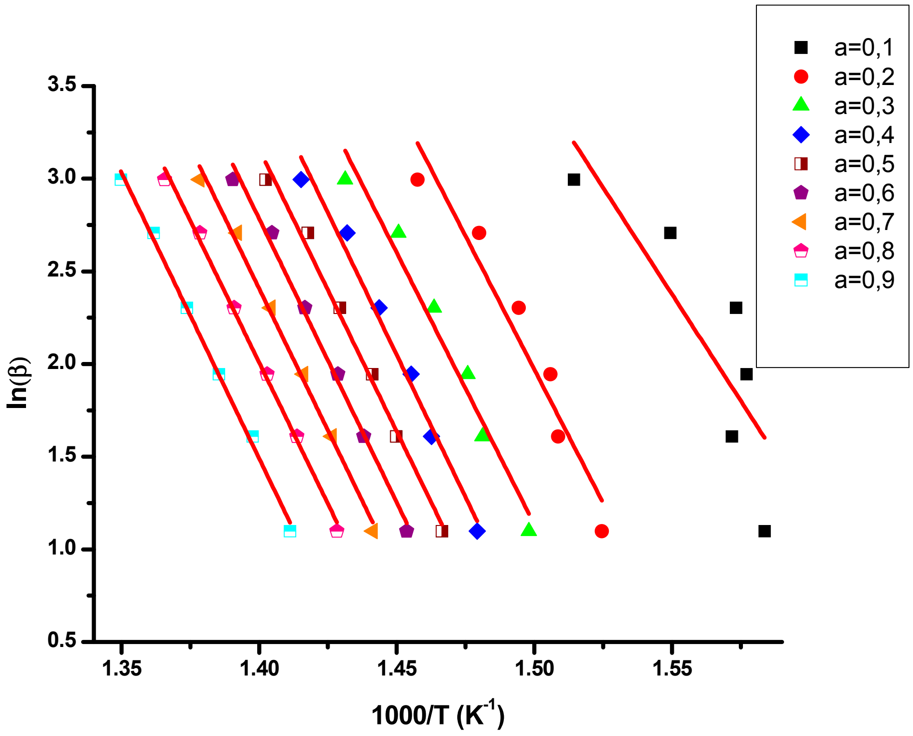

Figure S23. Ozawa-Flynn-Wall plots lnβ *vs.* 1/T for the sample 20/80P at different heating rates.

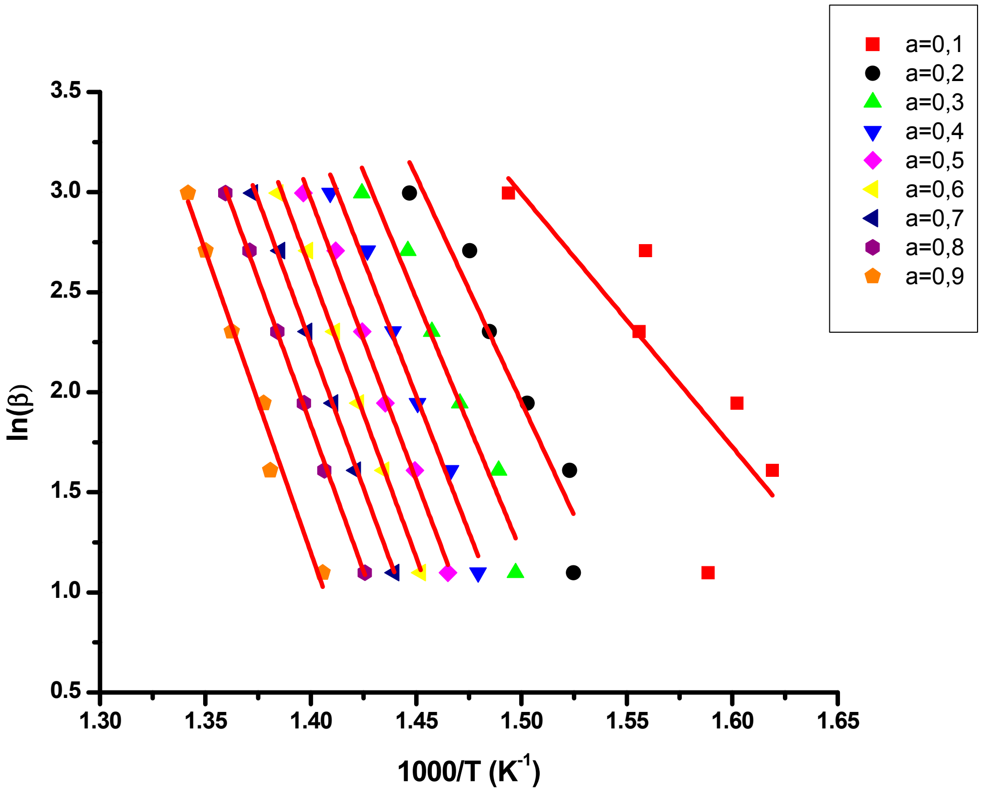

Figure S24. Ozawa-Flynn-Wall plots lnβ *vs.* 1/T for the sample 40/60P at different heating rates.

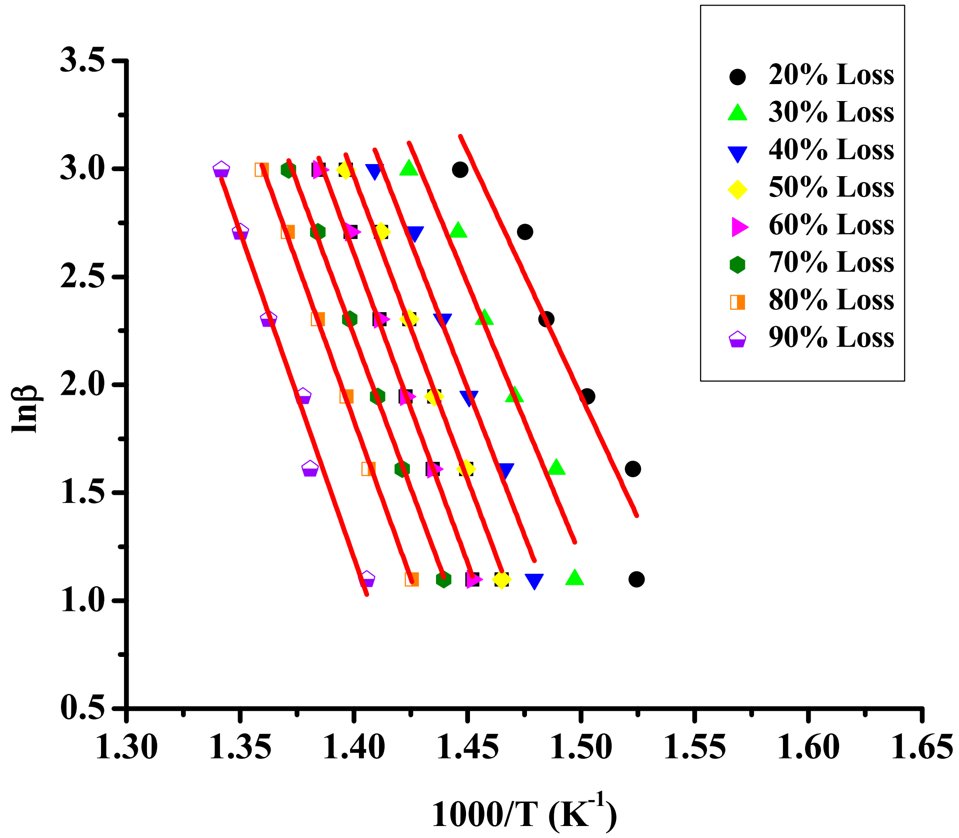

Figure S25. Ozawa-Flynn-Wall plots lnβ *vs.* 1/T for the sample 50/50P at different heating rates.

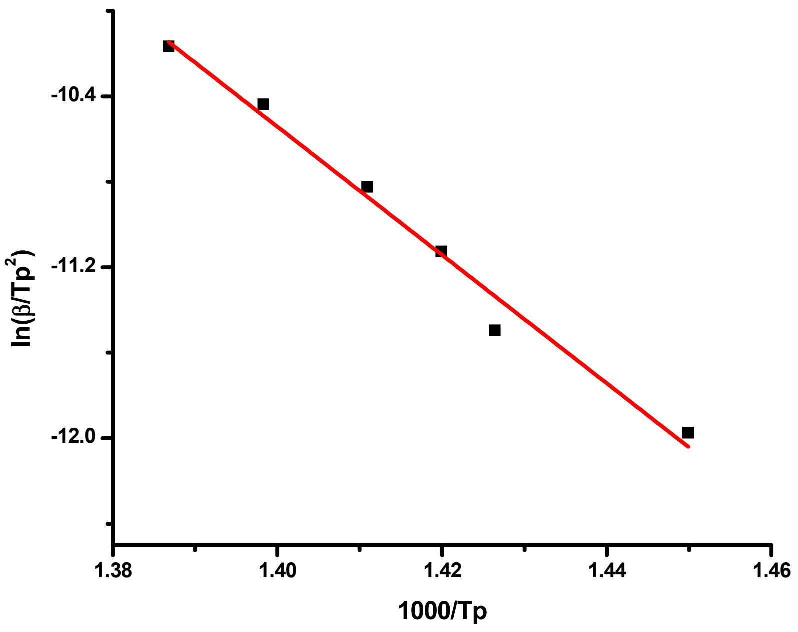

Figure S26. Kissinger plot $\ln(\beta/T_p^2)$ *vs.* $1000/T_p$ for sample 40/60P.

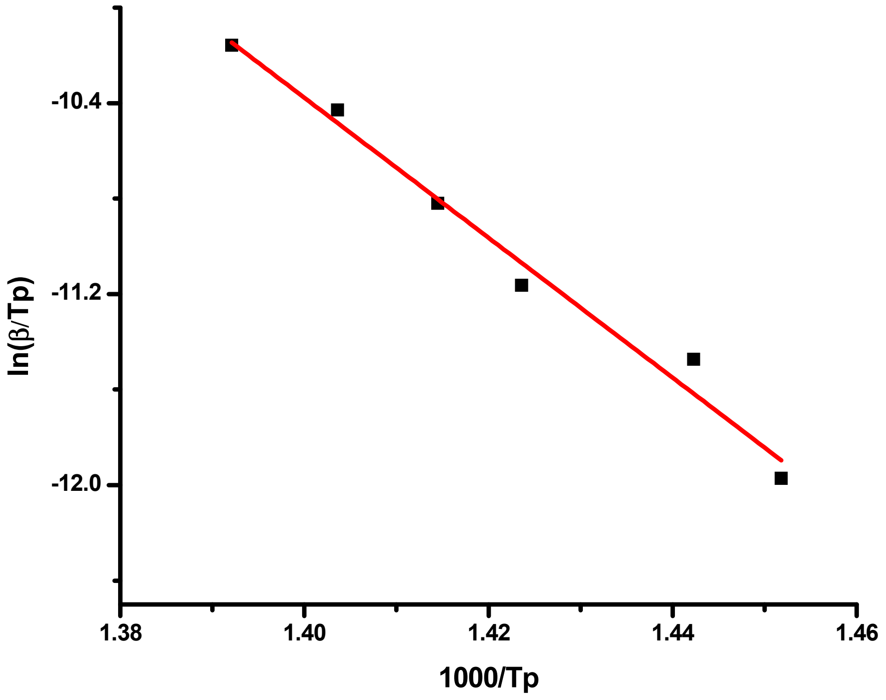

Figure S27. Kissinger plot ln(β/T_P^2) *vs.* 1000/T_P for sample 60/40P.

Table S1. TGA results for the homopolymers and the PNBE-*co*-PCP copolymers at 3 °C/min.

Sample	Start (°C)	Finish (°C)	Peak (°C)
**PCP**	294.19	467.90	426.45
**PNBE**	305.08	528.89	411.70
**80/20**	276.60	487.14	409.77
**60/40**	284.83	481.28	408.84
**50/50**	305.91	485.38	422.40
**40/60**	305.62	503.72	402.03
**20/80**	320.95	480.74	419.18

Table S2. TGA results for the homopolymers and the PNBE-*co*-PCP copolymers at 5 °C/min.

Sample	Start (°C)	Finish (°C)	Peak (°C)
**PCP**	306.83	477.24	435.00
**PNBE**	312.74	547.50	415.69
**80/20**	303.53	509.13	425.36
**60/40**	294.68	497.15	417.01
**50/50**	345.02	485.12	427.26
**40/60**	329.70	550.24	410.89
**20/80**	328.61	485.66	428.03

Table S3. TGA results for the homopolymers and the PNBE-*co*-PCP copolymers at 7 °C/min.

Sample	Start (°C)	Finish (°C)	Peak (°C)
**PCP**	315.63	480.54	438.90
**PNBE**	323.13	532.18	417.98
**80/20**	329.92	490.98	433.59
**60/40**	303.14	500.33	421.00
**50/50**	305.36	503.10	429.74
**40/60**	286.47	551.88	420.74
**20/80**	323.68	485.66	430.27

Table S4. TGA results for the homopolymers and the PNBE-*co*-PCP copolymers at 10 °C/min.

Sample	Start (°C)	Finish (°C)	Peak (°C)
**PCP**	317.49	499.99	446.82
**PNBE**	302.89	548.59	421.78
**80/20**	304.08	509.13	436.33
**60/40**	304.53	507.00	427.39
**50/50**	314.38	502.08	435.17
**40/60**	293.58	561.73	422.89
**20/80**	323.13	499.34	434.99

Table S5. TGA results for the homopolymers and the PNBE-*co*-PCP copolymers at 15 °C/min.

Sample	Start (°C)	Finish (°C)	Peak (°C)
**PCP**	321.67	495.93	454.04
**PNBE**	334.08	548.05	429.00
**80/20**	311.23	516.82	445.22
**60/40**	310.00	515.21	432.67
**50/50**	313.11	517.50	439.06
**40/60**	297.41	529.99	432.29
**20/80**	348.85	497.15	439.50

Table S6. TGA results for the homopolymers and the PNBE-*co*-PCP copolymers at 20 °C/min.

Sample	Start (°C)	Finish (°C)	Peak (°C)
**PCP**	318.92	504.18	460.24
**PNBE**	339.55	526.70	440.45
**80/20**	312.33	527.27	447.62
**60/40**	308.91	526.70	444.38
**50/50**	334.72	513.63	446.76
**40/60**	313.83	527.25	433.38
**20/80**	349.95	504.27	448.31

Table S7. TGA results for the PNBE-*co*-PCP copolymers prepared in the presence of PPh_3 at 3 °C/min.

Sample	Start (°C)	Finish (°C)	Peak (°C)
**80/20P**	335.08	460.61	418.95
**60/40P**	339.11	460.04	418.49
**50/50P**	318.96	465.80	415.77
**40/60P**	329.32	466.95	422.13
**20/80P**	310.99	461.47	416,61

Table S8. TGA results for the PNBE-*co*-PCP copolymers prepared in the presence of PPh_3 at 5 °C/min.

Sample	Start (°C)	Finish (°C)	Peak (°C)
**80/20P**	336.81	472.71	423.95
**60/40P**	340.26	468.67	423.04
**50/50P**	329.32	464.64	420.31
**40/60P**	334.51	470.40	428.05
**20/80P**	335.26	467.52	422.13

Table S9. TGA results for the PNBE-*co*-PCP copolymers prepared in the presence of PPh_3 at 7 °C/min.

Sample	Start (°C)	Finish (°C)	Peak (°C)
**80/20P**	369.06	477.31	432.14
**60/40P**	341.42	476.74	430.77
**50/50P**	351.78	473.86	429.41
**40/60P**	352.36	474.42	431.23
**20/80P**	335.08	475.01	427.59

Table S10. TGA results for the PNBE-*co*-PCP copolymers prepared in the presence of PPh_3 at 10 °C/min.

Sample	Start (°C)	Finish (°C)	Peak (°C)
**80/20P**	304.56	491.22	434.87
**60/40P**	302.84	483.07	429.41
**50/50P**	303.99	488.25	433.96
**40/60P**	313.78	491.13	435.78
**20/80P**	310.32	488.25	433.96

Table S11. TGA results for the PNBE-*co*-PCP copolymers prepared in the presence of PPh_3 at 15 °C/min.

Sample	Start (°C)	Finish (°C)	Peak (°C)
**80/20P**	313.20	506.68	443.06
**60/40P**	305.14	503.80	441.24
**50/50P**	302.84	498.04	439.42
**40/60P**	308.02	500.92	442.15
**20/80P**	315.50	498.62	440.33

Table S12. TGA results for the PNBE-*co*-PCP copolymers prepared in the presence of PPh_3 at 20 °C/min.

Sample	Start (°C)	Finish (°C)	Peak (°C)
**80/20P**	304.56	508.41	446.69
**60/40P**	332.20	502.26	448.97
**50/50P**	308.02	500.92	445.33
**40/60P**	325.87	503.22	448.06
**20/80P**	306.27	501.29	445.19
